# Using Large Language Models to Address Contextual Questions in Systematic Reviews

**DOI:** 10.1002/cesm.70060

**Published:** 2026-02-26

**Authors:** Susanne Hempel, Kimny Sysawang, Haley K. Holmer, Erin Tokutomi, Suchitra Iyer, Zhen Wang, Edi Kuhn, Mohammad Hassan Murad

**Affiliations:** ^1^ Southern California Evidence Review Center University of Southern California Los Angeles California USA; ^2^ Scientific Resource Center Portland VA Research Foundation Portland Oregon USA; ^3^ Evidence‐based Practice Center Program Agency for Healthcare Research and Quality Rockville Maryland USA; ^4^ Mayo Clinic Evidence‐Based Practice Research Program, Mayo Clinic Rochester New York USA

**Keywords:** artificial intelligence, context, large language models, systematic reviews

## Abstract

**Objectives:**

Systematic evidence reviews (SERs) produced by the U.S. Agency for Healthcare Research and Quality (AHRQ) Evidence‐based Practice Center (EPC) Program use contextual questions to provide context and background information on the topic. There is currently no standardized approach to address contextual questions in systematic reviews. This study explored the use of publicly available large language models (LLMs) in addressing contextual questions.

**Study Design:**

Using a set of 20 published and 5 yet to be published SERs, we selected one contextual question per report and used it as a prompt to elicit answers from an LLM (ChatGPT, Bard, Claude, or Perplexity). Two independent reviewers rated the results using a priori established evaluation criteria (https://osf.io/4k3cu/), comparing the response in the SER to LLM‐generated responses. The study was guided by six research questions addressing feasibility, validity of content, validity of structure, mistakes, congruence between responses, and incremental validity of using LLMs to address contextual questions.

**Results:**

Using minimal prompt engineering produced relevant responses and documented the feasibility of LLM‐generated answers to contextual questions. Responses differed in content and format and are not reproducible (e.g., LLMs update regularly), but LLMs were able to produce articulate, clinically plausible, and well‐structured responses. We detected few factual errors, contradictions, and no instance of suspected bias, but citations supporting LLM‐generated responses could often not be produced or could not be verified (‘confabulations’). Congruence with human generated responses varied, with LLM‐generated responses providing more background on the topic and SERs providing more nuanced answers in response to the contextual question. Results regarding incremental validity were mixed and may depend on the tool.

**Conclusion:**

LLMs are potentially helpful in addressing contextual questions in systematic reviews but human expertise remains essential for using the generated information in a meaningful way.

## 1 Introduction

Clinical decision‐making often requires contextual information in addition to comparative effectiveness findings answering the key questions of the systematic reviews. To address this need, some systematic evidence reviews (SER), such as those produced for the Agency for Healthcare Research and Quality (AHRQ) Evidence‐based Practice Center (EPC) Program [1], the Department of Veterans Affairs (VA) Evidence‐based Synthesis Program (ESP) [2], and U.S. Preventative Services Task Force (USPSTF) [3] SERs include contextual questions. SERs from these federal government programs inform clinical practice guidelines, healthcare policies, and research needs. Contextual questions differ from the key questions of the SER in that they help to provide background information on the SER topic, such as the natural history, prevalence and risk groups, variations in current clinical practice, access to care, available resources for patients, or frequently used approaches.

Aspects of the SER topic may emerge as candidates for contextual questions when the end‐users (i.e., policymakers, clinicians) need information to frame the findings of the SER. Information to address contextual questions is often gathered through targeted literature searches, authoritative surveys and published reviews, or expert input. Answers regarding contextual questions are usually included as part of the introduction or discussion section and narratively linked to the SER findings. Objectives resemble textual reviews addressed in JBI (formally known as the Joanna Briggs Institute, an organization known for its comprehensive methodologies and guidance on conducting systematic reviews) [4]. Textual evidence can be opinions, narratives, or policies, and may include clinical wisdom from health professionals, input from consumer and consumer representatives, or consensus guidelines from expert bodies. However, there is no standardized approach or guidance to address contextual questions in SERs.

With the rise of generative artificial intelligence (AI), large language models (LLMs) are increasingly being used in scientific writing; potential use cases include text editing, information compilation, and idea elaboration [5]. These models are trained on vast knowledge databases, and the tools can be prompted to answer a wide range of knowledge questions in a matter of minutes. By drawing on existing general knowledge and being able to follow concrete instructions, the LLM‐generated response has the potential to quickly highlight important aspects to consider in crafting a response to the contextual question. Alternatively, AI may guide the editorial structure of answers to contextual questions and potentially serve as brainstorming and idea‐generating support.

For this study, we hypothesized that LLMs, prompted by the contextual question (e.g., ‘What is the natural history of untreated spinal cord compression in patients with cervical degenerative disease?’) [6] of a recent SER, would generate an answer that includes key details and accurate information on the topic. We used publicly freely available or commercial LLMs that do not require staff with expertise in prompt engineering, and we purposefully did not focus on refining prompts to optimize answers to approach the task in a realistic scenario that could be incorporated into the SER production process. To our knowledge, it is unclear what kind of administrative and logistic burden or potential advantages are associated with using LLM‐generated responses to address contextual questions. The objective was to address the following six research questions:

1. What is the feasibility of using LLMs to generate reasonable answers to contextual questions posed in SERs?
2. What is the validity of the content in the LLM‐generated answers?
3. What is the validity of the structure of the LLM‐generated answers?
4. What is the accuracy of the content and number of mistakes included in the LLM‐generated answers?
5. What is the congruence of LLM‐generated answers with human generated answers to contextual questions included in recently published SERs?
6. What is the potential incremental validity of incorporating LLM‐generated responses in the process of answering contextual questions in SERs?

## 2 Methods

The project followed a detailed protocol [7]. We used two datasets. The first was a set of 20 published AHRQ EPC SERs with contextual questions. We selected SERs in reverse order of publication. The earliest report in this dataset was published in 2019 and the latest in 2023 (see Appendix A). We recorded 54 contextual questions and abstracted the questions together with the answers provided in the report. Dataset two was a set of five EPC SERs with contextual questions yet to be published at the time the LLM answers were generated. We used the most recent draft version of the SER with answers pertaining to the contextual questions. The five reports addressed 12 contextual questions in total. The EPC Program produces about 33 SERs per year. Of these, 28% of topics relate to preventive clinical services to inform USPSTF recommendations.

We evaluated for each contextual question whether it was clearly a question about context (e.g., asking about background for the review topic) or a question that could be addressed as a key SER question (e.g., asking about the effects of an intervention). In addition, we rated the level of detail included in the contextual question. The SERs were written by systematic review methodologists collaborating with subject matter experts. The contextual questions included in both datasets were answered by humans in the published or unpublished SER, without the help of LLMs or AI support. In a first exploratory evaluation, we examined the feasibility of using the exact contextual question posed in the SER as the LLM prompt; primarily aiming to determine whether the LLM could answer the question at all.

For dataset 1, we generated answers for all contextual questions with three LLMs (ChatGPT, Bard, Claude). We used ChatGPT (version 3) with training data up to October 2019, Bard (now replaced by Gemini) with training data up to early 2023, and Claude (version 2) with training data up to early 2023. The LLMs were known and available to the research team; no other consideration guided the selection of tools. We randomly selected one contextual question per report and one LLM answer using a random number generator.

From dataset 2, we randomly selected one contextual question per SER and posed the question to one of four LLMs (ChatGPT, Bard, Claude, Perplexity). The fourth LLM (Perplexity) was introduced due to its potential ability to generate scientific citations. We used Perplexity (version 2.27.2) with training data up to early 2023 for all five contextual questions in addition to the randomly selected answer generated by ChatGPT (version 3), Bard, or Claude (version 2). We tested whether models could produce citations, and we added a prompt to include citations. To standardize responses, we added the instruction to respond in paragraph format (rather than in bullet points or with text divided by subheadings).

We examined the feasibility of LLMs in generating reasonable answers to contextual questions posed in recent SERs (research question 1). We also recorded process information, such as whether the contextual questions could be asked as stated or whether prompt modifications were necessary, how much time was spent evaluating each answer, and which resources were used to fact‐check the LLM‐generated answer.

To answer research questions relating to the validity, accuracy, congruence, and incremental validity of the LLM‐generated answer, we determined evaluation criteria a priori. We evaluated the validity of the LLM‐generated answers based on the presented clinical information (research question 2). We also assessed whether the LLM‐generated answer may be skewed towards particular patient populations or interventions [8]. The validity of the structure of the LLM‐generated answers (research question 3) was assessed with the following question: is the overall impression of the LLM‐produced structure comprehensive and does it address diverse aspects of the topic? We rated the validity on a 5‐point rating scale ranging from the semantic differential of 1 (structure does not make sense) to 5 (compelling structure).

To establish content accuracy (research question 4), we recorded the number of contradictions, factual errors, and “confabulations,” and specified each identified instance. An example of a contradiction would be a situation where the first part of the LLM answer contradicts a later part. Factual errors were described as the LLM‐generated answer getting the direction of effects wrong or misstating the facts. “Confabulations” were defined as the LLM fabricating incorrect information. The human‐crafted responses in the SER were considered the reference standard for the answer content. The contextual questions were often complex with broad‐ranging topics, and to ensure consistency, we used the SERs as the main source of information to evaluate the LLM‐generated answer. Where information stated in the LLM‐produced answer could not be found in the SER, we used other AHRQ reports or online sources such as Google search, PubMed, UpToDate, Centers for Disease Control and Prevention (CDC) data, clinicaltrials. gov, and CoPilot AI for fact‐checking.

We also rated the overall congruence of the LLM‐generated answer with the answer to the contextual question in the SER (research question 5). We used a rating scale that ranged from 1 (does not match, e.g., states irrelevant information) to 5 (a good match and LLM contains the key points).

We determined the potential incremental validity of incorporating LLMs in the SER production process (research question 6) by recording the number of additional details that appeared to be a valid point in the context of the SER topic that was included in the LLM‐generated answer but that was not included in the SER. We recorded all instances of specific additions.

Two independent raters, both highly familiar with contextual questions and SERs, evaluated the LLM‐generated answers. We determined the agreement between the raters for this initial, independent rating, using absolute agreement, weighted kappa, and mean absolute differences. In a second step, both raters discussed their ratings and reconciled scores where discrepancies were detected. We computed the mean, standard deviation (SD), mode, and range across reconciled ratings.

## 3 Results

The assembled datasets are shown in Table S1. Throughout, there were considerable differences in the types of information sought by contextual questions across SERs. There was variation in the level of detail included in the contextual question (e.g., some contextual questions specified the population, intervention, or setting). Out of 25 included contextual questions, 8% were flagged as not detailed and potentially hindering meaningful LLM responses. Despite the label, some of the contextual questions were judged to be more typical of an effectiveness question, or key question in the SER, rather than purely addressing contextual information (and potentially could have been addressed with standard systematic review methodology). We did not detect systematic differences associated with the characteristics of the contextual questions. Table 1 documents the results of the ratings discussed below.

**Table 1:** Evaluation of LLM‐generated answer to contextual questions.

Report	Overall impression of LLM^a^	Bias^b^	Number of contradictions^c^	Number of factual errors^d^	Number of hallucinations^e^	Contradictions, factual errors, and hallucinations^f^ in LLM‐generated answers	Congruence between LLM and report answer^g^	Additional details in LLM answer^h^	Specification of additional details included in LLM‐generated answer	Time to evaluate LLM answer
Behavioral Interventions for Migraine Prevention: A Systematic Review^1^	5	No	1	0	3	The SER response focuses on the trials that included caregivers of kids with migraine, and said not much was available for adult care givers/spouses of adult atients. I didn't find the studies by Bond, Lipton or the SR mentioned.	2.5	Yes	Rater 1: LLM cited a systematic review which when available, would be better than citing individual studies. The SER cited individual studies.	50–60 min
Cervical Degenerative Disease Treatment^2^	4	No	1	0	0	Lack of quantitative data.	1	No	N/A	20–30 min
Counseling and Behavioral Interventions for Healthy Weight and Weight Gain in Pregnancy: A Systematic Review^3^	4.5	No	0	0	0	N/A	4	Yes	Rater 1: The two answers are very similar except that the SER answer referred to a specific source and provided estimates. One thing that LLM added was about the increase in the risk of neural tube defect, which was not addressed in the SER answer, and I verified this with outside sources such as PMID 34219595. Rater 2: Some info on neural tube defects and long term childhood outcomes, although that was not really the question (infant outcomes).	15–30 min
Effectiveness of Telehealth for Women's Preventive Services^4^	4.5	No	0	0	0	N/A	1	No	N/A	15–20 min
Integrated and Comprehensive Pain Management Programs: Effectiveness and Harms^5^	5	No	0	0	0	N/A	4	Yes	Rater 1: LLM answer is much more helpful for end users and is more pleasing to a reader. The LLM answer has good underpinnings and seems to be collated from various guidelines and resources. Rater 2: The LLM response is pretty generic. But for a naïve reader, the info could be useful. The SER has more info in the background. Didn't answer the question.	15–30 min
Long‐Term Health Outcomes in Obstructive Sleep Apnea: A Systematic Review^6^	5	No	0	0	0	N/A	5	Yes	Rater 1: LLM text included more verbiage and explanation, although this does not appear to be very substantive. Rater 2: Yes, hormone treatment, hypoglossal nerve stimulation to improve sleep and some additional explanatory text.	10–30 min
Management of Postpartum Hypertensive Disorders of Pregnancy^7^	5	No	0	0	0	N/A	4	Yes	Rater 1: The LLM answer is more comprehensive and better structured than the SER answer. However, it was not referenced (whereas the SER answer cited a specific study). The LLM answer also tried to provide a rationale and information about disparities with follow up care. Rater 2: Yes, it answered some aspects of the question more completely about how SDOH may impact access to good health and healthcare, baseline vulnerability of the populations, and brings up American Indian, an important subgroup. It doesn't provide data from studies that examined the link between social determinants of health and hypertensive disorders of pregnancy.	10–30 min
Measures for Primary Healthcare Spending^8^	2.5	No	0	0	0	N/A	3	No	N/A	15–60 min
Models of Care That Include Primary Care for Adult Survivors of Childhood Cancer: A Realist Review^9^	4.5	No	0	1	0	Discrepancy between the SER response (Despite the potential value of these resources, there is also evidence that they currently have limited reach and effectiveness) and LLM.	1	Yes	Rater 1: SER answer did not provide much whereas LLM answer was quite helpful. LLM answer was not referenced and did not provide association measures. LLM answer seems like a “pamphlet” given to patients. Rater 2: No, in fact it gives a very unclear response.	25–30 min
Partial Breast Irradiation for Breast Cancer^10^	5	No	0	0	0	N/A	3	Yes	Rater 1: LLM text has more explanation and justification, which we do not expect from the SER, which is mostly based on existing studies. Rater 2: The LLM response adds additional background info, such as cosmesis, but that may be addressed by the SER in other sections. Again, a summary, but doesn't answer the exact question of what studies have addressed and demonstrated.	25–30 min
Pre‐Exposure Prophylaxis for the Prevention of HIV Infection: A Systematic Review^11^	5	No	0	0	0	N/A	3	Yes	Rater 1: LLM answer is similar to SER but it doesn't reference specific studies (5 observational studies exist) and it does not provide specific quantitative estimates. Rater 2: Yes, it answered some aspects of the question more completely factors affecting adherence in greater detail, although a tad repetitive.	20–30 min
Prehabilitation and Rehabilitation for Major Joint Replacement^12^	4.5	No	0	0	0	N/A	1	Yes	Rater 1: LLM answer was more useful because it attempted to answer the question albeit using indirect evidence and extrapolation. Rater 2: Yes, LLM rambles a bit, but gives the sources of the costs associated with the interventions.	10–30 min
Psychosocial and Pharmacologic Interventions for Disruptive Behavior in Children and Adolescents^13^	3	No	0	0	0	N/A	1	Yes	Rater 1: The randomized LLM answer avoided disparities and filled with generic text to please the user. Perplexity answer addressed it, but with less specificity than SER answers. Rater 2: Info about conduct disorder and oppositional defiant disorder.	25–60 min
Respectful Maternity Care: Dissemination and Implementation of Perinatal Safety Culture to Improve Equitable Maternal Healthcare Delivery and Outcomes^14^	5	No	0	0	0	N/A	4.5	Yes	Rater 1: The concept of recognizing that childbirth is a natural process and not a medical condition was identified by LLM, not by the SER. Conversely, the component of continuity of care was in the SER answer, but not in LLM. Rater 2: LLM version was easier to read and understand.	10–30 min
Screening and Prevention of Dental Caries in Children Younger Than Age Five Years: A Systematic Review^15^	4	No	1	1	0	“Silver diamine fluoride (SDF) has become a valuable tool in pediatric dentistry, particularly for arresting existing cavities in children.” vs “If proven effective in preventing new cavities, SDF could become a significant addition to the pediatric dental toolkit.” Unclear why the specific trial was described and it was not on 3‐year olds but in elementary school children.	3	Yes	Rater 1: LLM answer points out an ongoing trial in children age 3, and does not point out the trial in those at 6 years old (mentioned by the SER). I found some indirect evidence (PMID 37789300 and PMID 36682908) that could have been leveraged in both answers (as indirect evidence). Rater 2: No, confusing structure. The info about clinical trials seems incorrect; there are several ongoing trials and the comparators vary.	20–40 min
Screening for Breast Cancer: A Comparative Effectiveness Review^16^	5	No	0	0	0	N/A	3.5	No	N/A	10–30 min
Screening for Chlamydial and Gonococcal Infections: A Systematic Review Update^17^	5	No	0	0	0.5	For chlamydial infections, research has shown that the prevalence rate among partners of individuals diagnosed with chlamydia can be as high as 60%–75%. Similarly, the prevalence rate of gonococcal infections among partners of patients diagnosed with gonorrhea is also significant, ranging from 20%–35% in various studies.	3	No	N/A	25–30 min
Screening for Depression, Anxiety, and Suicide Risk in Adults: A Systematic Evidence Review^18^	5	No	0	1	0	Slope bias, potentially not relevant, not an error but not relevant to the topic.	3	Yes	Rater 1: LLM answer is more eloquent and helpful. It addresses internal and external validity and explains test bias. The LLM response is more satisfying to the user and appears more comprehensive. Rater 2: LLM version was easier to read, although it may have conflated concepts of screening and diagnosis.	30 min
Screening for Glaucoma in Adults: A Systematic Review^19^	5	No	0	0.5	0	Unable to confirm that visual loss doesn't affect visual acuity.	3.5	Yes	Rater 1: Close answers but SER answer seemed to be derived from a specific study whereas the LLM answer seems to be derived from variable sources. Rater 2: LLM doesn't have stats but seems to be more cautious in underscoring benefit from intraocular pressure reduction on visual acuity.	20–30 min
Screening for Syphilis Infection in Nonpregnant Adults and Adolescents: A Targeted Evidence Update^20^	5	No	0	0	0	N/A	4.5	Yes	Rater 1: LLM provided info on cost and when to use the tests in relation to prevalence, which was not addressed in the SER answer. Rater 2: Less jargon and more readable.	15–30 min
Screening, Referral, Behavioral Counseling, and Preventive Interventions for Oral Health in Adults: A Systematic Review^21^	4	No	0	0	0	N/A	2	Yes	Rater 1: The LLM answer is much more comprehensive and useful to end users, although it seems to rely mostly on indirect evidence. It seems that the LLM tried to make extrapolation of data from other context, which seems quite reasonable. Unfortunately, the lack of citations makes it challenging to judge certainty in the LLM answer. Rater 2: Adds some valuable info around factors for success of interventions. They seem plausible, but difficult to verify sources.	15–30 min
Screening, Referral, Behavioral Counseling, and Preventive Interventions for Oral Health in Children and Adolescents Ages 5 to 17 Years: A Systematic Review^22^	4	No	0	0	0	N/A	2	Yes	Rater 1: The LLM answer is much more comprehensive and useful to end users, although it seems to rely mostly on indirect evidence. It seems that the LLM tried to make extrapolation of data from other context, which seems quite reasonable. Unfortunately, the lack of citations makes it challenging to judge certainty in the LLM answer. Rater 2: No. Doesn't address effectives or disparities. Assumes effectiveness, which could be considered an error, perhaps not a hallucination.	15–30 min
Statin Use for the Primary Prevention of Cardiovascular Disease in Adults: A Systematic Review^23^	3.5	No	0	0	0	N/A	2.5	No	N/A	10–30 min
Strategies for Integrating Behavioral Health and Primary Care^24^	5	No	0	0	0	N/A	3.5	Yes	Rater 1: The LLM answer was easier to read and more user friendly because it actually discussed barriers and facilitators, whereas the SER answer emphasized the methods used to come up with an answer (causal diagram, framework) with less actual discussion of barriers and facilitators. The LLM answer was not referenced.	20–60 min
Trauma Informed Care^25^	4	No	0	0	0	N/A	3	No	N/A	25–60 min

*Note:* SER references are shown in the appendix.

Abbreviations: LLM large language model; min, minutes; N/A, Not applicable, not available; SDOH, social determinants of health; SER, systematic evidence review; vs, versus.

^a^ Is the overall impression of the LLMs produced structure comprehensive and addressing diverse aspects? Rated on a 5‐point rating scale ranging from 1 (structure doesn't make sense) to 5 (compelling structure).

^b^ Does the LLM answer appear to have introduced bias with regard to health disparities or equity? If yes, please specify.

^c^ Content accuracy: What is the number of contradictions (e.g., the first half of the answer contradicts something it is saying in the second half of the answer)?

^d^ Content accuracy: What is the number of factual errors (e.g., the LLM got the direction of effects wrong or got confused about the facts)?

^e^ Content accuracy: What is the number of hallucinations (e.g., the LLM makes something up i.e. incorrect, including references that do not exist)?

^f^ What were the contradictions, factual errors, and hallucinations?

^g^ What is the congruence with answers to contextual questions published in recent systematic evidence review reports (e.g., does it match)? Rated on a 5‐point rating scale ranging from 1 (does not match e.g., states irrelevant information) to 5 (good match e.g., LLM picks up on key points).

^h^ Does the LLM add anything important, are there valid details that the systematic evidence review report doesn't have?

^i^ If yes, please specify.

^j^ How long did it take you to evaluate the LLM answer (e.g., looking up facts)?

### 3.1 Feasibility of LLM Generating Reasonable Answers to Contextual Questions Posed in Systematic Reviews

Access to the LLMs was easy to set up and all produced responses within seconds after providing the prompt. LLMs were able to generate reasonable sounding and relevant answers for all evaluated contextual questions, that is, answers were articulate and grammatically correct and appeared clinically plausible. Using the exact contextual question as stated in the SER as the prompt appeared to be successful in answering the specific contextual questions.

The LLM‐generated responses differed in word count, editorial structure, content, and ability to include correct citations. Except for the model Perplexity, the evaluated LLMs did not provide text with scientific citations. Three out of the four LLMs did not produce citations even when prompted to do so, and some even explicitly stated that the LLM is unable to produce citations (one LLM indicated copyright reasons for being unable to access journal publications).

In dataset 1, the amount of time spent evaluating the LLM‐generated answer and fact‐checking ranged from 10 min to 30 min per SER. For dataset 2, the amount of time spent ranged from 15 min to 60 min per SER.

### 3.2 Validity of the Content in Answers Produced by the LLM

An initial review of the responses had indicated that the LLMs generated reasonable answers across diverse contextual questions and SER topics. We also found that the content was built from varied sources and not simply generated by detecting the original SER that included the contextual question and copying the SER answer. Forty‐five percent of the SERs in dataset 1 were published in the public domain before the date the LLM was trained. Dataset 2 consisted of reports that were not published yet, avoiding any possibility of LLMs using the SER as a source for the LLM‐generated answer. Responses to contextual questions from published as well as unpublished SERs were deemed appropriate.

### 3.3 Validity of the Structure of the LLM Generated Answers

Most LLM‐generated answers were rated a 5 on a scale from 1 (structure does not make sense) to 5 (compelling structure). The range in individual ratings was 2 to 5. Rater agreement is documented in Table S2 and was 0.55 for this global rating. The mean validity rating reconciled by the two raters was 4.48 (SD 0.70). The ratings indicated that LLM‐generated answers read well, were well‐structured, and contained no grammatical errors. Neither rater detected instances of bias towards a particular population or intervention in the response.

### 3.4 Content Accuracy and Mistakes in LLM‐Generated Answers

The independent expert raters both evaluated most answers to be error free (absolute agreement ranged from 0.90 for contradictions, 0.80 for factual errors, and 0.85 for ‘confabulations’). The reconciliation process between the human raters detected a few potential content accuracy issues.

A contradiction was detected in an answer to a contextual question regarding dental caries in children in dataset 1 (the LLM‐generated answer stated that silver diamine fluoride has become a valuable tool in pediatric dentistry, and that it could become a significant addition to the pediatric dental toolkit depending on the outcome of ongoing trials). The discussion of the LLM‐generated responses confirmed a factual error in a LLM‐generated response from dataset 1 associated with a SER on screening for glaucoma (the LLM‐generated answer included a statement that visual field loss does not affect visual acuity).

Neither rater detected errors or contradictions in dataset 2. However, two cited individual studies and one cited systematic review (all three included in the same LLM‐generated answer to a contextual question), could not be verified and we classified this as a “confabulation.” While the cited study authors had published on the topic, no matching publication in that publication year was found. The LLM did not provide a link to the cited publications. The raters initially only searched PubMed to verify the citations, but a research librarian subsequently searching other research databases was also unable to locate an existing publication that matched these references (i.e., citations “Bond, 2021;” “Lipton, 2009;” Kröner‐Herwig and Gassmann (2019);” see Table S1).

### 3.5 Congruence With Answers to Contextual Questions Published in Recent Systematic Evidence Review Reports

The congruence of responses to contextual questions generated by LLM compared to those in the SER was limited. The mean rated congruence of LLM‐answer and SER after rater reconciliation was 2.86 (SD 1.19) across all SERs on a scale ranging from 1 (do not match) to 5 (good match, sources pick up the same points). While the LLM‐generated responses provided more general background information on the topic, the SERs had more relevant and nuanced answers addressing the contextual question. Both raters also indicated that both sources included important aspects in the answer to the contextual question.

### 3.6 Potential Incremental Validity of Incorporating LLMs in the Process of Answering Contextual Questions in Systematic Evidence Review Reports

Responses varied by SER and rater regarding the question of whether the LLM‐generated responses added important details that the SER answer did not include. LLM‐generated responses were described as easier to read, and resembled lay summaries in SERs that redact specific numbers. LLM‐generated answers tended to provide broader information on the SER topic, for example, adding background information regarding screening in general instead of strictly answering the specific question regarding the context for screening addressed in the SER. Only one of the LLMs was able to produce scientific citations; and these citations were also often generic, that is, although relevant to the broader topic, they were not specific or underpinning distinct points. LLM‐generated answers conveyed confidence in the answer and rarely acknowledged uncertainty, requiring literature reviewers to check and contextualize information.

## 4 Discussion

This project documents favorable results regarding the feasibility of using LLM tools to generate answers to contextual questions posed in systematic reviews but also highlights important limitations.

We found that the LLM‐generated answers were generally polished and well structured. In addition, raters indicated repeatedly that the LLM‐generated content provided a user‐friendly response. However, raters also pointed to instances where they found the LLM‐generated content to be not very substantive, i.e., providing generic information about the overarching topic that did not directly address the contextual question. Our findings align with previous studies using LLMs for scientific writing [9]. A study using LLMs to create plain language summaries to support accessibility of evidence review findings indicated promising results in terms of stylistic features of responses, but the authors concluded that the summaries likely require human input to ensure accuracy, comprehensiveness, and appropriate nuances of interpretation. Similarly, a study that used the JBI tool for text and opinion to evaluate the content and structure of 1000‐word reviews generated by ChatGPT found that the LLM‐generated content was well‐articulated and logical but it lacked depth [10].

Based on our experiences, the LLM‐generated answers could produce a useful initial draft which may function as an idea‐generating support tool that provides authors with a starting point upon which to expand and deepen the connection between the contextual issue and the evidence in the SER. This procedure potentially facilitates consistency in how contextual questions are addressed in systematic reviews. LLMs can quickly and systematically extract information related to the context from a wide range of information sources. The scalability of LLMs' ability to handle large volumes of research data makes it less topic dependent. Previous attempts to automate text in systematic reviews have focused on producing structured templates. For example, researchers have developed an add‐on for the Cochrane Collaboration software RevMan (now replaced by RevMan Web) to auto‐generate abstract, results, and discussion sections (RevMan HAL) [11]. The authors stressed that the text is generated from highly structured data, it can use generic and topic‐unspecific text for content that exists in variations in all systematic reviews.

LLMs are a black box—we give them input and they produce results, but the process is not well understood. In addition, LLMs do not produce reproducible results, and LLMs are constantly retrained and new versions are released. The research reported in this paper is not reproducible, because none of the LLM versions that were used are available anymore. In addition, there is a lack of transparency in that the training data for LLMs is typically not well described, i.e., it is less clear what data the answers draw from. LLMs are unable to replicate answers specific to the user and timestamp. Although, contextual questions typically involve stable factors, hence as long as the model has access to the same dataset, the answers will be similar. Training data for LLMs are collected up to a specific date. As a result, a model's performance and understanding is limited to information available up to that time period and may not reflect recent developments, trends, or changes in the topic of interest. This is important to consider when evaluating the model's accuracy and applicability in real‐world settings as it will not account for relevant post‐cutoff events, emerging patterns, or newly available data. LLMs have different version dates because they undergo regular updates and improvements to enhance performance, capabilities, and accuracy, and it is critical to report the version. Transparency and reproducibility of responses generated by LLMs are widespread concerns [12, 13].

To improve consistency and transparency, research will need to establish procedural and reporting guidelines for how LLMs can support contextual questions, similar to JBI guidance for textual reviews [14]. The JBI guide suggests establishing a structured and pre‐determined framework for the evidence included in the review. Synthesis should follow guidance for qualitative evidence to generate a set of statements that adequately represents the identified information, and reporting should include the number of textual evidence identified, retrieved, appraised, excluded, and included. Similarly, as a minimum, authors need to report the version of the LLM, the date to determine which training dataset the information is based on, and what exact prompts were used to generate the information. Guidance should be harmonized with other best practices and recommendations, including the RAISE initiative (Responsible AI use in Evidence SynthEsis) [15, 16].

In this study, we found very few instances of mistakes such as factual errors or contradictions. However, provision of citations underpinning evidence statements proved to be problematic. Most evaluated LLMs were unable to provide any citation in the format of scientific references. When prompted to provide citations, these LLMs explicitly stated that they cannot add citations. Only one of the evaluated LLMs was able to produce citations at all. However, the model typically cited relevant but very generic sources, often to make generic statements on the topic instead of answering the specific contextual question. Producing too generic answers may be an overarching issue with LLMs. Furthermore, in one instance, the LLM model produced three citations that could not be verified. This suggests that while the LLM can generate a reasonable structure for the response to contextual questions, systematic reviewers will still need to manually identify research citations underpinning the content, because the LLMs are either unable to provide citations or can produce citations that do not exist.

Furthermore, we used the original contextual questions as prompts and questions varied in their level of detail. To optimize LLM‐generated responses, the level of detail in the question may need to be adjusted because the details effectively function as prompts. Prompt engineering is a crucial step in harnessing the power of LLMs. It involves carefully crafting prompts that provide the model with the necessary instructions to generate accurate and relevant responses [17, 18]. However, this introduces a risk of overfitting—the prompts are refined so they work well with specific training data, but not necessarily on a larger corpus. By providing clear and concise prompts, researchers may significantly improve the quality and efficiency of LLM‐assisted systematic reviews. Another effective technique for enhancing LLM performance is few‐shot learning [19]. This approach involves providing the model with a few examples of the desired input‐output pairs during the prompt engineering process. By exposing the model to these examples, it can learn to generalize and generate more accurate responses to novel questions within the specific area of interest of the systematic review. LLM performance may be further improved by redefining prompts to elicit a pre‐specified response for the intended audience [20]. Furthermore, Low‐Rank Adaptation (LoRA) allows for the fine‐tuning of LLMs on specific tasks with reduced computational resources [21]. By training only a small subset of the model's parameters, this would enable researchers to adapt LLMs to their unique requirements, leading to improved performance and tailored responses.

We used LLMs specifically to generate answers to contextual questions. Throughout the systematic review process, context may play a role in topic refinement, protocol development, data extraction, and interpretation of results. Understanding the needs of the end‐user is essential for determining the approach used to answer contextual questions. Booth et al. [22] have summarized tools and methods used to account for context in systematic reviews, and note that context is important when planning a review as well as when synthesizing review findings. The use of LLMs to address contextual questions may be of most value when refining the scope of a planned systematic review or at the report writing stage after the review has been completed. For topic refinement, the training sets of LLMs may quickly clue systematic reviewers into the nuances of the topic (e.g., decisional dilemmas, interventions of interest; important evidence gaps). With report writing, LLMs may readily assist authors with structuring contextual information in light of the systematic review findings.

While LLMs are rapidly evolving und the research base continues to develop, LLMs have been utilized for systematic reviews in various ways, most importantly for literature searches, screening studies for relevance, and abstracting data [23]. However, using LLMs in systematic reviews has methodological limitations due to the need for transparency in conducting systematic reviews. Our research showed that using the original contextual questions as prompts produced relevant responses, but the responses differed across LLMs in word count, editorial structure, content, and ability to produce real citations. Some LLMs were better in answering the contextual question than others in that they provided more specificity by addressing components of the contextual question with dedicated paragraphs specific to each aspect of the question. If LLMs are used for research, it is crucial that it is understood that the exact results cannot be replicated and that the information sources are not fully transparent.

Finally, risk of bias is a concern for human reviewers as well as LLM models. Human reviewers, when interpreting context may inadvertently introduce and amplify cognitive biases. LLMs, with appropriate training on sufficiently representative datasets, can potentially apply a more objective and consistent approach to identifying and integrating context, improving the overall quality of the evidence synthesis. Although we did not identify instances of bias in our study, we suggest that this phenomenon be addressed in more detail in future studies.

Our study had multiple limitations that should be considered when interpreting the data. First, we used only a small set of reports, contextual questions are not a core aspect of systematic reviews, and LLMs develop rapidly. Furthermore, we used the SER answer as a gold standard and compared the congruence of the LLM‐generated answer to the answer given in the SER. This procedure was necessary for pragmatic reasons given that large range of clinical topics that is addressed in the included SERs for which otherwise content experts would have had been engaged to validate the LLM‐generated answer. However, it is a limitation given that we did find that LLM‐answers also added unique contributions and the lack of similarity with the SER did not necessarily mean that the answer was factually incorrect. It should also be noted that some of the assessments relied on the judgement of the raters, such as the rating of reasonable sounding answers. While we used two independent raters, only two raters in total assessed all reports, and the assessment used scales designed for the purpose of this study rather than validated instruments. In addition, all contextual questions were taken from AHRQ EPC SERs and future research needs to determine whether findings are similar for other contextual questions. And as discussed, LLMs are a quickly changing field and the versions of LLMs used for this study have since been updated, raising reproducibility concerns.

Future direction of this study to optimize the LLM's performance could include providing the full‐text of research studies included in the SER as the information source to generate answers to contextual questions. Provision of a set of studies may help to fine‐tune the LLM rather than solely relying on the general knowledge of the LLM's training data. Additionally, as noted earlier, refining the prompts could further frame the LLM's answers to the topic of the systematic review. Furthermore, we tracked the time to produce LLM‐generated answers and resources needed for the fact‐checking process. However, we did not assess time and resources needed for human authors to produce an answer to a contextual question. Hence a critical comparison is still missing, and future studies should directly compare time and resources needed for both processes to pinpoint the value of adding LLMs to the process producing SERs with context questions.

Given the lack of a standardized procedure to answering contextual questions in systematic reviews, the use of LLM tools provides a potential useful step and may add some consistency to the process. Ultimately, while LLMs can be useful in helping to answer contextual questions, human expertise is still essential for using and interpreting information in meaningful ways.

## Author Contributions

**Susanne Hempel:** conceptualization, investigation, funding acquisition, writing – original draft, methodology, writing – review and editing, formal analysis, project administration, data curation, supervision, resources. **Kimny Sysawang:** investigation, writing – original draft, methodology, formal analysis, data curation. **Haley K. Holmer:** conceptualization, investigation, writing – review and editing, methodology. **Erin Tokutomi:** investigation, methodology, data curation, resources. **Suchitra Iyer:** conceptualization, investigation, methodology, writing – review and editing, data curation. **Zhen Wang:** conceptualization, investigation, methodology, writing – review and editing. **Edi Kuhn:** investigation, methodology, writing – review and editing. **Mohammad Hassan Murad:** conceptualization, investigation, funding acquisition, methodology, data curation.

## Conflicts of Interest

The authors declare no conflicts of interest.

## Supporting information

Appendix revised.

## Data Availability

The data that support the findings of this study are openly available in Open Science Framework at https://osf.io/4k3cu, reference number 4k3cu. Data is available in the Open Science Framework record osf. io/4k3cu for this project.
