# Supplementary material for: Using Large Language Models to Address Contextual Questions in Systematic Reviews
Source: Cochrane Evid Synth Methods. 2026 Feb 26;4(2):e70060. doi: 10.1002/cesm.70060 (PMC12948247; doi:10.1002/cesm.70060)
Supplement: Supplementary file 1 — Appendix revised. [file CESM-4-e70060-s001.docx]

Appendix

**Appendix Table 1: Contextual Qustions, Human and LLM-generated Answers**

| **SER Title** | **Contextual Question with AI Prompt** | **SER Answer** | **Randomly Selected LLM-generated Answer**^a^ | **Perplexity AI**^b^ |
| --- | --- | --- | --- | --- |
| Behavioral Interventions for Migraine Prevention: A Systematic Review  Link: https://www.pcori.org/research-results/2022/behavioral-interventions-migraine-prevention-systematic-review  Report date: September 2024  Contextual questions per report: 2  Level of detail: Detailed  Context or effectiveness question: Effectiveness | What evidence is available on the benefits of behavioral preventive treatments for children and adults with migraine that include intervention components targeting caregivers (e.g., parents, spouses, and other key support people)? Respond in paragraphs. | Behavioral treatments for migraine prevention that include components targeting caregivers are primarily focused on the parents and caregivers of adolescents and/or children with migraine (not caregivers of adults with migraine). Parents and caregivers are often involved in treatment and undoubtedly play a vital role in its management, particularly for elements that are self-guided, such as those that are Web-based. Notably, having a child or children with migraine often results in caregiver burden and increases parental stress; therefore, incorporating caregivers into treatment may also benefit their well-being directly (through targeted intervention components) or indirectly (because of improvements experienced by their child). Two well-known examples of behavioral interventions for pediatric migraine with caregiver components are the “Headstrong” and “MIPAS-Family” studies (MIPAS is an acronym for Migraine Patient Seminar Program). The Headstrong RCT evaluated the effectiveness of a self-guided CD-ROM program containing cognitive-behavioral self-management strategies for preventing pediatric headaches and additionally evaluated headache-related disability and QOL. Parents were given a manual containing directions on how to use the Headstrong program, their role in the intervention (e.g., how to assist with homework assignments, how to complete headache diaries), lesson overviews, and technical assistance information in case their child had problems running the program. Participants and their parents completed all measures independently. Compared with an educational control, Headstrong resulted in lower pain severity at post-treatment and less migraine-related disability at 3 months postintervention, by child and parent report, respectively. No significant differences were observed for headache frequency or QOL. The MIPAS-Family RCT compared the impact of a multi-modal behavioral education and training program with a combined biofeedback treatment. Parents were given four training sessions that spanned three modules: diagnostic, education, and behavioral training. Child and parent components alternated to facilitate parents becoming co-trainers who helped employ the techniques at home. Both intervention groups showed significant improvements concerning headache intensity and duration. The RCT found no significant differences in the main headache parameters between the two groups. After the treatments, children were less disturbed by their headaches in the domains of school, homework, and leisure time. An additional example is an older controlled study that explored the use of parent-mediated pain behavior management strategies as an adjunct to the biofeedback treatment of children with migraine. Children receiving parent-mediated pain management guidelines evidenced significantly greater reductions in headache frequency, were more likely to experience clinically significant improvements, were more likely to be headache-free, and demonstrated better adaptive functioning during treatment and at 3-month follow-up. Generally, studies like these suggest that behavioral treatments for pediatric/adolescent migraine benefited from using a holistic, caregiver-centered approach. | There is limited but promising evidence on the benefits of behavioral preventive treatments for migraine that incorporate intervention components targeting caregivers of both children and adults with migraine. For children with migraine, several studies have examined the effectiveness of cognitive-behavioral therapy (CBT) interventions that involve parents or caregivers. These interventions typically educate parents on migraine management strategies, such as identifying and addressing triggers, implementing lifestyle modifications, and providing support during migraine attacks. The inclusion of parents or caregivers in the treatment has been found to enhance treatment adherence, improve coping skills, and reduce migraine frequency and disability in children. A systematic review by Kröner-Herwig and Gassmann (2019) concluded that involving parents in psychological interventions for pediatric migraine can lead to significant reductions in headache frequency, intensity, and disability. For adults with migraine, the evidence on caregiver-involved interventions is more limited. However, some studies have explored the potential benefits of including spouses or partners in behavioral migraine treatments. These interventions often focus on enhancing communication, problem-solving, and stress management skills within the couple or family unit. A pilot study by Lipton et al. (2009) found that a couples-based CBT intervention led to significant reductions in migraine disability and improved marital satisfaction compared to a control group. Another study by Bond et al. (2021) reported that a family-based CBT intervention for adults with migraine resulted in reduced migraine frequency and improved quality of life. While the evidence is still emerging, these findings suggest that incorporating caregivers in behavioral preventive treatments for migraine can be beneficial for both children and adults. By equipping caregivers with knowledge and coping strategies, these interventions can promote better treatment adherence, reduce migraine burden, and enhance overall well-being for individuals with migraine and their support systems. | Behavioral preventive treatments for migraine that include components targeting caregivers, such as parents, spouses, and other key support people, have shown promising results for both children and adults. These interventions often incorporate cognitive-behavioral therapy (CBT), biofeedback, relaxation techniques, and mindfulness-based strategies, which are designed to reduce the frequency, severity, and duration of migraine attacks, as well as improve overall quality of life. Evidence for Behavioral Interventions: A meta-analysis by the U.S. Headache Consortium concluded that relaxation training, thermal biofeedback combined with relaxation training, electromyographic biofeedback, and cognitive-behavioral therapy are effective options for migraine prevention. These therapies can be used alone or in combination with pharmacologic treatments to enhance clinical outcomes for patients suffering from migraines[1]. Role of Caregivers in Behavioral Interventions: Caregivers play a critical role in the success of these behavioral interventions. For children, involving parents in the treatment process can help reinforce the techniques learned during therapy sessions and provide emotional support. For instance, parents can assist in maintaining a consistent routine for relaxation exercises or biofeedback sessions, which can lead to better adherence and outcomes. Similarly, spouses and other key support people can help adults with migraines by providing encouragement and ensuring that stress-reducing activities are integrated into daily life[2]. Benefits of Behavioral Interventions: Behavioral interventions have several benefits over pharmacologic treatments, particularly in reducing the risk of side effects. Nonpharmacologic therapies such as progressive muscle relaxation, biofeedback, and mindfulness-based interventions have demonstrated efficacy in reducing migraine frequency and pain intensity, as well as improving overall well-being. These interventions are particularly valuable for children and adolescents, for whom pharmacologic options are limited and often come with significant risks[5]. Systematic Reviews and Ongoing Research: Ongoing research and systematic reviews are continually updating the evidence base for behavioral interventions in migraine prevention. The Patient-Centered Outcomes Research Institute (PCORI) and the Agency for Healthcare Research and Quality (AHRQ) are conducting a systematic review to provide updated guidelines on the management of migraines, including the role of behavioral interventions. This review aims to synthesize recent evidence and inform clinical practice, highlighting the importance of integrating : caregivers into the treatment process[5]. In summary, behavioral preventive treatments for migraine that involve caregivers have shown significant benefits in managing migraine symptoms and improving patients' quality of life. These interventions offer a valuable alternative or complement to pharmacologic treatments, particularly for populations such as children and adolescents who may be more vulnerable to medication side effects. Citations: [1] https://www.aafp.org/pubs/afp/issues/2019/0101/p17.html [2] https://americanheadachesociety.org/topic/behavioral-treatment/ [3] https://www.ncbi.nlm.nih.gov/books/NBK507873/ [4] https://effectivehealthcare.ahrq.gov/products/behavioral-interventions-migraine-prevention/protocol [5] https://www.pcori.org/research-results/2022/behavioral-interventions-migraine-prevention-systematic-review |
| Cervical Degenerative Disease Treatment  Link: https://effectivehealthcare.ahrq.gov/sites/default/files/product/pdf/cervical-degenerative-protocol.pdf  Report date: June 2023  Contextual questions per report: 2  Level of detail: Detailed  Context or effectiveness question: Contextual | What is the natural history of untreated spinal cord compression in patients with cervical degenerative disease? Respond in paragraphs. | The natural history of degeneration of the cervical spine progressing to nonmyelopathic spinal cord compression (NMSCC) and ultimately CSM is a continuum of disease that remains poorly understood. Untreated spinal cord compression is most studied in the context of CSM. There is a subset of patients with spinal cord compression found on imaging who are asymptomatic. A recent systematic review by Nouri et al (2022) found the prevalence of asymptomatic spinal cord compression in healthy volunteers to be 24.2 percent (range 5.3% to 59%). A small series by Martin et al (2018) looking at 20 asymptomatic patients with MRI evidence of spinal cord compression revealed that 2 (10%) developed symptoms of myelopathy at a median followup of 21 months. The largest prospective study evaluating the transition from NMSCC to CSM by Bednarik et al (2008) revealed that among 199 patients enrolled with NMSCC, 8 percent developed CSM at 1-year followup and 22.6 percent of patients developed CSM at median followup of 44 months (range 1-12 years). Factors found to independently predict the development of myelopathy in a multivariate analysis included presence of radiculopathy, spinal cord cross-sectional area and compression ratio. CSM is the leading cause of spinal cord dysfunction among adults worldwide. The pathogenesis of CSM is due to both mechanical and neuropathic changes to the spinal cord and blood spinal cord barrier generated by compression on the spinal cord. The compressed cervical spinal cord is subjected to chronic hypoxic conditions due to dysfunction of endothelial cells as well as flattening and consequent loss of surrounding vessels. While the natural history of CSM in patients varies greatly, it is generally thought of as a progressive disorder. This was confirmed in a recent systematic review181 that found moderate evidence from small prospective and retrospective studies that the proportion of patients who deteriorate by at least 1 point in the JOA scale ranged from 20 to 60 percent. It is important to point out that these studies did not consider the minimal detectible difference to define deterioration, which is >1 point based on reliability studies. The overall lack of large, well designed and controlled studies evaluating the natural history of untreated spinal cord compression in patients with CDDs impairs clinicians’ ability to counsel patients. A recent clinical practice guideline provided by AO Spine suggested that either surgery or clinical observation are reasonable initial treatment options in mild CSM (e.g., mJOA score greater than or equal to 15). Shimomura et al evaluated prognostic factors for deterioration of patients with CSM treated nonoperatively. Their prospective study included 56 patients with mild CSM, 11 (20%) had clinical deterioration over a mean followup period of 35.6 months. Age, gender, followup period, developmental or dynamic canal factors (e.g., canal size of < 12mm) of cervical spine on plane lateral radiographs, presence of high intensity of the cord on T2 weighted MRI and circumferential spinal cord compression on axial MRI were all evaluated as possible predictors for progression of myelopathy. However, they found the only predictive factor was presence of circumferential spinal cord compression on axial MRI (adjusted OR 26.6, 95% CI 1.7 to 421.5). 186 More studies are needed to better define the natural history of untreated spinal cord compression in the setting of degenerative changes along with predictors of progression. | Untreated spinal cord compression in patients with cervical degenerative disease follows a progressive and potentially debilitating natural history. Initially, individuals may experience intermittent symptoms such as neck pain, stiffness, and occasional numbness or tingling in the arms. These symptoms often arise due to the compression of nerve roots or spinal cord structures by degenerated discs, bone spurs, or thickened ligaments in the cervical spine. Over time, as the compression persists and worsens, the symptoms tend to become more frequent, severe, and persistent. As the compression on the spinal cord increases, patients may develop neurological deficits, including weakness, sensory disturbances, and loss of coordination in the upper extremities. This progression can significantly impair activities of daily living and reduce quality of life. Furthermore, untreated spinal cord compression can lead to more severe complications, such as gait disturbances, difficulty with balance, and impaired fine motor skills. In some cases, individuals may experience bowel or bladder dysfunction, which can further impact independence and overall well-being. Without intervention, the natural history of untreated spinal cord compression can ultimately result in irreversible neurological damage and permanent disability. As the compression persists, there is a risk of developing progressive myelopathy, characterized by worsening motor and sensory deficits, spasticity, and even paralysis. These advanced stages of cervical myelopathy can be profoundly disabling and may necessitate extensive rehabilitation or assistive devices to manage daily activities. Given the potential for significant morbidity and loss of function, timely diagnosis and appropriate management are essential in patients with cervical degenerative disease and spinal cord compression. Early intervention, which may include conservative measures such as physical therapy, medications, or interventional procedures, aims to alleviate symptoms, slow disease progression, and preserve neurological function. In cases where conservative measures are inadequate or symptoms continue to worsen, surgical decompression may be necessary to relieve pressure on the spinal cord and prevent further neurological deterioration. Overall, proactive management strategies are crucial to optimize outcomes and improve the long-term prognosis for individuals with untreated spinal cord compression due to cervical degenerative disease. | N/A |
| Counseling and Behavioral Interventions for Healthy Weight and Weight Gain in Pregnancy: A Systematic Review  Link: https://www.uspreventiveservicestaskforce.org/home/getfilebytoken/VW-5qn-usE92nF_5Bee9aW  Report date: May 2021  Contextual questions per report: 3  Level of detail: Detailed  Context or effectiveness question: Contextual | What is the association between high pre-pregnancy BMI and risk of adverse maternal and infant outcomes? Respond in paragraphs. | A key result of the LifeCycle Project-Maternal Obesity and Childhood Outcomes Study was that prepregnancy BMI was more strongly associated with adverse maternal and infant outcomes than GWG. Numerous observational studies have demonstrated an association between elevated BMI at pregnancy onset and adverse perinatal outcomes. A 2017 umbrella review of 156 meta-analyses of cohort studies evaluated the association between adiposity and multiple obstetric and gynecological outcomes; evidence was strongest for elevated prepregnancy BMI and increased risk of cesarean delivery (BMI less than 25 vs. greater than 30; OR, 2.00 [95% CI, 1.87 to 2.15]), preeclampsia (BMI less than 25 vs. 25-30; OR, 1.70 [95% CI, 1.60 to 1.81]), and antenatal depression (BMI less than 25 vs. greater than 30; OR, 1.48 [95% CI, 1.32 to 1.66]; p<0.001 for all comparisons). The evidence was highly suggestive for associations between elevated prepregnancy BMI and GDM, LGA, macrosomia, stillbirth, postpartum hemorrhage, and preterm birth. There was a dose-dependent association between increasing degree of maternal obesity and increased maternal morbidity. A large (N=226,958) population-based cohort study from Canada examined the relationship between prepregnancy BMI and adverse pregnancy outcomes. The study estimated a 10 percent reduction in prepregnancy BMI would be associated with at least a 10 percent lower risk of preeclampsia, gestational diabetes, preterm delivery, macrosomia, and stillbirth. For example, women with a BMI of 36 kg/m2 would have a 10 percent lower risk of these complications than women with a BMI of 40 kg/m2. Larger declines in prepregnancy BMI (20% to 30%) were necessary to reduce risks of cesarean, shoulder dystocia, NICU stay 48 hours or longer, and in-hospital newborn mortality. Prepregnancy BMI was not associated with risk of postpartum hemorrhage requiring intervention, severe maternal morbidity or maternal mortality, or spontaneous preterm delivery before 32 weeks of gestation. | High prepregnancy BMI (Body Mass Index) is strongly associated with an increased risk of adverse maternal and infant outcomes. Maternal obesity, classified typically as a BMI of 30 or higher, has been linked to a variety of complications during pregnancy, labor, and delivery. One of the significant concerns is the elevated risk of gestational diabetes mellitus (GDM), which can lead to larger-than-average babies (macrosomia), increasing the likelihood of cesarean sections and associated complications. Additionally, mothers with high BMI are more susceptible to hypertensive disorders, including preeclampsia, which can have severe implications for both maternal and fetal health, necessitating early delivery and increasing neonatal intensive care unit (NICU) admissions. For the infant, the ramifications of high maternal BMI can begin in utero and extend well beyond birth. Infants born to obese mothers are at higher risk for congenital anomalies, including neural tube defects and cardiovascular abnormalities. The risk of preterm birth, either spontaneous or medically indicated, is also elevated, leading to potential long-term health issues for the child. Moreover, these children are more likely to experience metabolic disturbances, such as insulin resistance and higher adiposity in childhood, predisposing them to obesity and type 2 diabetes later in life. The adverse outcomes are not only limited to the immediate perinatal period but can also affect the long-term health and well-being of both mother and child. For mothers, the challenges posed by high prepregnancy BMI include a greater likelihood of retaining pregnancy weight gain, which can perpetuate or worsen obesity, thus increasing the risk for chronic conditions such as cardiovascular disease and type 2 diabetes. Consequently, the intergenerational transmission of obesity risk factors becomes a concern, highlighting the critical need for effective interventions to manage and reduce obesity before and during pregnancy to improve both maternal and infant health outcomes. | N/A |
| Effectiveness of Telehealth for Women’s Preventive Services  Link: https://effectivehealthcare.ahrq.gov/sites/default/files/product/pdf/cer-256-telehealth-women-preventive-services.pdf  Report date: June 2022  Contextual questions per report: 1  Level of detail: Detailed  Context or effectiveness question: Contextual | What guidelines, recommendations, or best practices have been developed for the design and use of telehealth and virtual health technologies for women for any clinical conditions, including patient preferences, patient choice, patient engagement, and implementation in low-resource settings? Respond in paragraphs. | Although there are no formal guidelines for telehealth delivery of preventive services, guidance by leading professional organizations for the use of telehealth services can be found. Many organizations have recommended that access to family planning services should be available via telehealth, especially during the COVID-19 pandemic. Several groups, including those who support the use of telehealth for family planning services, have recommended reducing barriers to contraceptive access during the COVID-19 pandemic through a variety of mechanisms. These include performing new patient contraceptive visits via telehealth, prescribing multi-month contraception at reduced or no cost, providing counseling about postponing removal of LARC, prioritizing in-person contraceptive visits to placement of LARC while performing pre-procedural counseling via telehealth, training and offering self-administered injectable contraception, and utilizing pharmacist prescribed contraception. A proposed option to facilitate routine screening for IPV during the COVID-19 pandemic is to include telehealth via technology-enabled interventions, which has been shown to be preferred by IPV survivors in other contexts. IPV organizations have emphasized the importance of continued screening via telehealth visits with clinicians using trauma informed approaches. | Telehealth and virtual health technologies hold immense promise for women's healthcare. To ensure this potential is met, several key principles guide their design and use. First, patient preferences and choice are paramount. Telehealth consultations should foster open communication, where women understand the advantages and limitations of virtual care compared to in-person visits. This empowers them to make informed decisions about their healthcare delivery model. The design of telehealth platforms should also cater to individual needs. Some women might be comfortable with video calls, while others might prefer phone consultations or secure messaging. Offering multiple communication options ensures inclusivity. Patient engagement is another crucial aspect. Providing clear instructions on using the platform, troubleshooting tips, and information about what to expect during the virtual visit can significantly improve a woman's comfort level and participation. Additionally, interactive features like educational materials, appointment reminders, and secure messaging for follow-up questions can enhance communication and empower patients to be active participants in their healthcare. When considering implementation in low-resource settings, scalability and accessibility become critical factors. Telehealth solutions should be designed with bandwidth limitations and affordability in mind. Mobile-friendly platforms and features that work offline can significantly increase accessibility for women in these areas. Ideally, telehealth platforms should integrate seamlessly with existing electronic health record systems to ensure continuity of care and data accessibility for healthcare providers. Additionally, collaborations with local community centers or libraries can provide crucial access to technology and internet connectivity for women who might otherwise lack the resources for telehealth participation. By prioritizing patient preferences, engagement, and accessibility, telehealth can evolve into a valuable tool for expanding access to quality women's healthcare, even in areas with limited resources. | N/A |
| Integrated and Comprehensive Pain Management Programs: Effectiveness and Harms  Link: https://effectivehealthcare.ahrq.gov/sites/default/files/pdf/cer-251-integrated-pain-management.pdf  Report date: October 2021  Contextual questions per report: 2  Level of detail: Detailed  Context or effectiveness question: Contextual | What pain management models or mechanisms are most commonly used in clinical practice? Respond in paragraphs. | The current paradigm for pain care is the provision of selected individual treatments (e.g., medications) or services (e.g., physical therapy, psychological support) prescribed or recommended by a patient’s provider (primary care or specialty provider). | In clinical practice, pain management involves a variety of models and mechanisms aimed at addressing acute and chronic pain conditions effectively. One commonly used approach is the biopsychosocial model, which considers the interplay between biological, psychological, and social factors in pain perception and management. This model recognizes that pain is a complex phenomenon influenced by physical pathology, psychological factors such as mood and cognition, and social determinants such as culture, environment, and socioeconomic status. By addressing these multidimensional aspects of pain, clinicians can develop comprehensive treatment plans that incorporate pharmacological interventions, physical therapy, psychological support, and social interventions to optimize pain relief and improve patient outcomes. Pharmacological interventions are a cornerstone of pain management and encompass a wide range of medications targeting various mechanisms of pain transmission and modulation. Nonsteroidal anti-inflammatory drugs (NSAIDs), opioids, anticonvulsants, antidepressants, muscle relaxants, and topical agents are among the most commonly prescribed medications for pain relief. These medications may be used alone or in combination to provide analgesia, reduce inflammation, alleviate neuropathic pain, and improve functional status. However, clinicians must carefully balance the benefits of pain relief with the potential risks of adverse effects, tolerance, dependence, and misuse associated with certain medications, particularly opioids. Nonpharmacological pain management strategies play an increasingly important role in clinical practice and may be used alone or in conjunction with pharmacological interventions to enhance pain relief and functional outcomes. Physical therapy, including exercise, manual therapy, and modalities such as heat, cold, ultrasound, and electrical stimulation, can help improve mobility, strength, and flexibility, reduce muscle tension, and promote tissue healing. Cognitive-behavioral therapy (CBT) and other psychological interventions aim to modify maladaptive thoughts, emotions, and behaviors associated with pain, enhance coping skills, and promote self-management strategies. Additionally, complementary and alternative therapies, such as acupuncture, massage therapy, mindfulness-based stress reduction (MBSR), and relaxation techniques, may offer additional avenues for pain relief and symptom management. Multidisciplinary pain management programs represent an integrated approach to pain care that brings together a team of healthcare professionals with expertise in various disciplines, including pain medicine, physical therapy, psychology, rehabilitation, nursing, and social work. These programs offer comprehensive assessments, individualized treatment plans, and coordinated care to address the complex needs of patients with chronic pain conditions. By combining pharmacological, nonpharmacological, and psychosocial interventions within a collaborative care model, multidisciplinary pain management programs aim to optimize pain relief, improve functional outcomes, and enhance quality of life for patients living with chronic pain. In conclusion, pain management in clinical practice involves a variety of models and mechanisms aimed at addressing acute and chronic pain conditions comprehensively. These approaches encompass pharmacological interventions, nonpharmacological strategies, multidisciplinary care models, and the biopsychosocial model of pain management, which integrates biological, psychological, and social factors into treatment planning. By adopting a holistic approach to pain care that considers the unique needs and preferences of individual patients, clinicians can optimize pain relief, improve functional outcomes, and enhance overall quality of life for individuals living with pain. | N/A |
| Long-Term Health Outcomes in Obstructive Sleep Apnea: A Systematic Review  Link: https://www.ahrq.gov/sites/default/files/wysiwyg/research/findings/ta/sleep-apnea/sleep-apnea-report.pdf  Report date: December 2022  Contextual questions per report: 5  Level of detail: Detailed  Context or effectiveness question: Contextual | What treatment modalities for OSA are currently being marketed in the United States? What OSA treatments (experimental or approved) are currently being investigated in ongoing trials for patients as an alternative to CPAP? Respond in paragraphs. | Positive airway pressure therapy, namely, CPAP, is commonly considered to be a first-line treatment for OSA. Positional therapy, weight loss, and behavioral modifications are (often ancillary) interventions that do not require particular devices. Several oral appliances (e.g., mandibular advancement devices) have been cleared or approved by the FDA to be legally marketed in the U.S. Newer treatments include nasal expiratory positive airway pressure (EPAP) devices, oral pressure therapy, and implantable hypoglossal nerve stimulation devices. Hypoglossal nerve stimulation by means of implantable devices is being examined for patients with moderate to severe OSA. | Currently, there are several treatment modalities for obstructive sleep apnea (OSA) being marketed and available in the United States. The most commonly used treatment is continuous positive airway pressure (CPAP) therapy, which involves wearing a mask connected to a machine that delivers a steady stream of air to keep the airway open during sleep. In addition to CPAP, other treatment options being marketed include oral appliances, which are dental devices worn during sleep that reposition the jaw or tongue to keep the airway open, such as mandibular advancement devices and tongue-retaining devices. Positional therapy, involving the use of devices or positioning techniques to prevent patients from sleeping on their backs, is also available, as sleep apnea is often worse in the supine position. For some patients with specific anatomical obstructions, surgical interventions like uvulopalatopharyngoplasty (removal of excess tissue from the soft palate and throat) or maxillomandibular advancement (surgical repositioning of the jaw) may be recommended. Researchers are also investigating alternative treatments for OSA that are currently in various stages of clinical trials. One experimental treatment being studied is hypoglossal nerve stimulation, which involves implanting a device that electrically stimulates the hypoglossal nerve, controlling tongue movement, to prevent airway obstruction during sleep. Nasal expiratory positive airway pressure (nEPAP) devices, small disposable devices worn over the nostrils that create resistance during exhalation to help keep the airway open during inhalation, are also being explored. Several pharmacological interventions, including medications that target neurotransmitters or hormones involved in sleep regulation, are being investigated for their potential to improve OSA symptoms. Oropharyngeal exercises, specific exercises targeting the muscles of the tongue and throat, are being explored as a potential non-invasive treatment option for mild to moderate OSA. Given the strong association between obesity and OSA, various weight loss strategies, including lifestyle modifications, pharmacotherapy, and bariatric surgery, are being studied for their potential to improve OSA outcomes. While these alternative treatments show promise, many are still in the experimental or early clinical trial phases, and their long-term efficacy and safety are yet to be fully established. Ongoing research and clinical trials are crucial for evaluating the potential of these treatments as viable alternatives or adjuncts to CPAP therapy for patients with OSA. | N/A |
| Management of Postpartum Hypertensive Disorders of Pregnancy  Link: https://effectivehealthcare.ahrq.gov/sites/default/files/related_files/cer-263-postpartum-hypertensive-pregnancy.pdf  Report date: May 2023  Contextual questions per report: 1  Level of detail: Detailed  Context or effectiveness question: Contextual | How are race, ethnicity, and social determinants of health related to disparities in incidence and detection of hypertensive disorders of pregnancy (HDP), as well as access to care, management, follow-up care, and clinical outcomes in individuals with postpartum hypertensive disorders of pregnancy? Respond in paragraphs. | Data suggest that HDP is more common and more severe in Black women. A study of insurance claims concluded that 14 percent of severe maternal morbidity events occurred after the delivery hospitalization, most within the first 2 weeks after discharge. The adjusted odds of having a de novo severe maternal morbidity event were 1.69-fold higher in non-Hispanic Black women than in all other women. Racism, whether structural, institutional, or individual, is understood to be the root cause of the disparities in incidence, severity, and outcome, mediated by multiple levels of influence that impact structural and social determinants of health. Recent editorials argue that race is a poor proxy for human variation and such racially tailored care may worsen health inequities if effective therapies are withheld based on providers’ perception of patients’ race. | Race, ethnicity, and social determinants of health (SDOH) are deeply intertwined with disparities in hypertensive disorders of pregnancy (HDP). Black, American Indian/Alaska Native (AI/AN), and Hispanic women face a higher risk of HDP compared to white women. This can be due to genetics and pre-existing conditions like chronic hypertension and obesity, which are more common in these populations. SDOH significantly impact how HDP unfolds. Limited access to quality healthcare can leave pre-existing conditions undiagnosed or poorly managed, and delay prenatal care, all increasing HDP risk. Lower socioeconomic status can further disadvantage women, limiting access to healthy food and preventive care while elevating stress levels. Even environmental factors might play a role. These disparities extend beyond just the initial diagnosis. Minority women may struggle to access prenatal care or specialists, and might experience implicit bias from healthcare providers. Unconscious bias or lack of awareness about disparities can lead to less aggressive treatment for HDP complications. Additionally, they might be less likely to receive adequate follow-up care after delivery, increasing the risk of long-term health problems. These factors all contribute to poorer clinical outcomes for minority women with HDP, making them more susceptible to severe complications and even death. To address these disparities, a multi-faceted approach is needed. We need to expand access to quality healthcare, reduce costs, and eliminate transportation barriers. Medical training should address implicit bias and promote cultural competency. Finally, HDP prevention and management strategies should consider SDOH, focusing on early intervention and addressing risk factors specifically within under-served communities. Only by tackling these issues can we work towards ensuring equitable care for all pregnant women. | N/A |
| Measures for Primary Healthcare Spending  Link: https://effectivehealthcare.ahrq.gov/products/primary-healthcare-spending/protocol  Report date: May 2025  Contextual questions per report: 2  Level of detail: Detailed  Context or effectiveness question: Contextual | Is there any emerging consensus among experts in the field toward a standard or preferred method for assessment of primary care spending? Format in full sentences and paragraphs, avoid bullet points and bolded headers. | There are a small number of health services researchers and policy analysts who are experts in the area of primary care spending measurement. These experts call for standardizing how we estimate primary care spending. They recognize that a national standard would provide a common way of looking at primary care spending across all 50 States. This is needed because, as Koller points out, “measuring primary care spending rates has proven to be an effective means for focusing public attention on primary care” and “primary care spending rate is a reasonable measure of the relative priority that an entity places on primary care…” it can be easily understood and calculated. Other experts agree with Koller and note there is a growing concern about an increasingly specialist-oriented healthcare system. To strengthen the Nation’s primary care foundation requires that we are able to “meaningfully quantify current and future health system investment in primary care” and a standardized way for measuring this investment is needed. This is how policymakers and decisions makers use these measures; they draw attention to current investment levels, to implement policies to increase that investment, and to establish and monitor accountability for reaching primary care spending targets, if such a target has been established through State legislation. A smaller number of policymakers are tracking how increases in investment are leading to lower overall costs of healthcare. Rhode Island is the only State that tracked this and the foundation-generated report for California also looked at this for certain payers. There is no evidence of benchmarking across States or connecting primary care spending to health equity or health outcomes. Yet, there is little uniformity in defining primary care spending. “Assessing spending in a standardized way” would allow States “to meaningfully quantify current and future health system investment in primary care” and it would allow others to objectively compare primary care spending geographically and across payers and healthcare systems and promote transparency of overall investment in primary care. In terms of consensus for a standard preferred method of estimating primary care spending, experts agree that an integrated framework for estimating primary care spending must encompass claims- and nonclaims-based components of primary care spending. Experts also acknowledge that States need a full method for measuring primary care spending provided to them, and they suggest that providing this method to States may expedite adoption, promote standardized measurement, and facilitate cross State comparisons. Experts note that accomplishing this is possible. It will require national level leadership and investment that starts with a definition of high-quality primary care, a common definition of primary care spending, and a method for collecting the data needed to construct that estimate that minimizes burden. While there may be some opportunities to use State APCD and those assembled by voluntary State-level collaboratives might work, these databases lack some of the data elements needed to identify primary care spending. A national standard for measuring primary care may need to be developed as an auxiliary measure. This is because States will continue to have some different measurement methods that are tailored to their local environment. An auxiliary measure would produce one estimate of primary care spending using the same methods for all 50 States. The 11 States that are already measuring primary care spending could continue to do so using their definitions of primary care spending, many of which are legislatively mandated. These States would have access to both estimates, and States that have not yet constructed a primary care estimate would have one (the national standard) that they could choose to adopt. Since States are using claims and nonclaims data to produce State estimates, using the same data sources in the national estimate would encourage alignment, understanding and adoption of measurement methods by other States. | There's a growing acknowledgment among experts in the field of healthcare economics and policy regarding the importance of accurately assessing primary care spending. Primary care is considered a cornerstone of effective healthcare systems due to its focus on preventive care, early intervention, and holistic patient management. However, historically, there hasn't been a standardized method for assessing primary care spending, leading to challenges in accurately measuring its impact and allocating resources effectively. In recent years, there has been momentum towards establishing a standard or preferred method for assessing primary care spending. One emerging approach involves using a comprehensive definition of primary care that encompasses a range of services, including preventive care, chronic disease management, coordination of care, and patient education. By adopting a broader definition, experts aim to capture the full scope of primary care activities and their associated costs, providing a more accurate picture of investment in this critical area of healthcare. Another important aspect of standardizing primary care assessment is the development of robust measurement tools and data sources. This includes leveraging electronic health records (EHRs), claims data, and other administrative sources to track primary care spending over time. Additionally, efforts are underway to refine methodologies for distinguishing between primary care and specialty care spending within healthcare expenditure data, ensuring that investments in primary care are accurately identified and measured. Furthermore, there is increasing recognition of the need to assess primary care spending in relation to health outcomes and healthcare utilization. Simply measuring the amount of money allocated to primary care is insufficient; it's essential to understand how these investments translate into improved patient outcomes, reduced healthcare costs, and enhanced population health. By integrating outcome measures into primary care assessment frameworks, experts can better evaluate the value and effectiveness of primary care investments and inform future resource allocation decisions. Overall, while there may not yet be a fully standardized method for assessing primary care spending, there is a growing consensus among experts regarding the key components and principles that should underpin such an approach. By adopting comprehensive definitions, leveraging robust measurement tools and data sources, and incorporating outcome measures, policymakers and healthcare stakeholders can better understand the role of primary care in achieving the goals of a high-performing healthcare system. As efforts continue to evolve in this area, it's likely that consensus will continue to build around best practices for assessing and optimizing primary care spending. | spending has been identified as a significant barrier to effectively measuring and comparing investment in primary care across different health systems, states, and organizations. Several efforts have been made to move towards a more standardized approach. For instance, the Patient-Centered Primary Care Collaborative and the Robert Graham Center have highlighted the need for a consensus definition of primary care and a standardized method for calculating primary care spend. They argue that such standardization would allow for better benchmarking and comparisons across health plans, accountable care organizations, and states, as well as facilitate more robust research on the relationship between primary care investment and health outcomes[1]. Some progress has been made in developing frameworks for assessing primary care spending. The Primary Care Spend Model, proposed by some researchers, offers a tiered structure that allows for varied definitions of primary care while providing a unified framework for comparison. This model aims to accommodate different data limitations and international health system differences while still offering guidance for best accounting practices[4]. At the state level, there are examples of efforts to standardize primary care spending assessment. For instance, Rhode Island has implemented a new methodology for measuring primary care expenditures, which will be used for public reporting and enforcing compliance with commercial expenditure targets[5]. This suggests that some jurisdictions are moving towards more standardized approaches, even in the absence of a national consensus. Despite these efforts, significant variability remains in how primary care spending is defined and calculated. Estimates of the percentage of total U.S. healthcare spending on primary care range from 5.8% to 7.7%, with wide variation depending on the definition and methodology used[2]. This variability underscores the ongoing need for a more standardized approach. While a clear consensus has not yet emerged, there is a growing recognition among experts that reaching agreement on a standardized method for assessing primary care spending is crucial. Such a consensus would enable more accurate comparisons, inform policy decisions, and potentially support efforts to increase investment in primary care. As more researchers focus on this topic and more states pursue primary care investment policies, the momentum towards developing a consensus definition and measurement approach is likely to increase. Citations: [1] https://www.graham-center.org/content/dam/rgc/documents/publications-reports/reports/Investing-Primary-Care-State-Level-PCMH-Report.pdf [2] https://thepcc.org/sites/default/files/resources/PCPCC%20Consensus%20Recommendations%20on%20Primary%20Care%20Investment%20Aug%202018.pdf [3] https://nap.nationalacademies.org/resource/25983/Highlights_High-Quality%20Primary%20Care-4.23.21_final.pdf [4] https://www.ncbi.nlm.nih.gov/pmc/articles/PMC6626519/ [5] https://ohic.ri.gov/sites/g/files/xkgbur736/files/2023-12/Primary%20Care%20in%20Rhode%20Island%20-%20Current%20Status%20and%20Policy%20Recommendations%20December%202023.pdf |
| Models of Care That Include Primary Care for Adult Survivors of Childhood Cancer: A Realist Review  Link: https://effectivehealthcare.ahrq.gov/sites/default/files/product/pdf/models-care-adult-survivors-childhood-cancer-evidence-report.pdf  Report date: February 2022  Contextual questions per report: 4  Level of detail: Detailed  Context or effectiveness question: Effectiveness | What is the evidence of effectiveness of the different resources available for adult survivors of childhood cancer and their families or their care providers? Respond in paragraphs. | Answer not discussed. | Evidence of the effectiveness of resources available for adult survivors of childhood cancer and their families or caregivers is multifaceted, reflecting the complex needs of this population. Numerous studies have evaluated various supportive resources, including survivorship clinics, educational materials, peer support groups, and online resources, among others. These resources aim to address the unique physical, psychosocial, and informational needs of childhood cancer survivors and their families as they transition from pediatric to adult-centered care settings. Survivorship clinics, often housed within cancer centers or academic medical institutions, provide specialized care tailored to the long-term health needs of childhood cancer survivors. These clinics offer comprehensive health assessments, screening for late effects of cancer treatment, health education, and personalized survivorship care plans to promote optimal health outcomes and quality of life. Research suggests that survivorship clinics play a crucial role in improving healthcare utilization, promoting adherence to recommended follow-up care, and enhancing survivor and caregiver satisfaction with healthcare experiences. Educational materials, such as brochures, pamphlets, and online resources, provide valuable information on cancer survivorship, late effects of treatment, recommended health screenings, and strategies for self-care and symptom management. These materials empower survivors and their families to make informed decisions about their healthcare and enhance their ability to navigate the complexities of survivorship. Studies have shown that access to high-quality educational resources improves knowledge retention, promotes self-advocacy, and facilitates shared decision-making between patients, caregivers, and healthcare providers. Peer support groups and online communities offer opportunities for childhood cancer survivors and their families to connect with others who share similar experiences, challenges, and concerns. These support networks provide emotional validation, social support, and practical advice on coping with survivorship issues, fostering a sense of belonging and community among participants. Research indicates that engagement in peer support activities reduces feelings of isolation, enhances psychosocial well-being, and improves coping skills among survivors and their families. Additionally, interventions targeting caregivers of childhood cancer survivors, such as counseling, psychoeducation, and respite care services, have been shown to alleviate caregiver burden, improve caregiver mental health outcomes, and enhance overall family functioning. By addressing the unique needs of caregivers, these interventions help support the well-being of both survivors and their families, promoting resilience and adaptive coping strategies in the face of survivorship challenges. Overall, the evidence supports the effectiveness of a multifaceted approach to supporting adult survivors of childhood cancer and their families or caregivers, encompassing survivorship clinics, educational materials, peer support groups, and caregiver interventions. By providing comprehensive support across the survivorship continuum, these resources help promote optimal health outcomes, enhance quality of life, and foster resilience among childhood cancer survivors and their families as they navigate the challenges of survivorship. | N/A |
| Partial Breast Irradiation for Breast Cancer  Link: https://effectivehealthcare.ahrq.gov/sites/default/files/related_files/cer-259-partial-breast-irradiation.pdf  Report date: January 2023  Contextual questions per report: 1  Level of detail: Detailed  Context or effectiveness question: Contextual | In adult women with early-stage breast cancer, to what extent does financial toxicity differ between partial and whole breast irradiation? Respond in paragraphs. | We did not identify any studies that explicitly addressed the construct of financial toxicity, defined as subjective or objective financial distress and hardship experienced by patients due to cancer-related (or anticipated) treatment. However, we identified studies that addressed various closely related concepts, such as direct nonhealthcare costs (e.g., transportation to receive care) and indirect costs (e.g., loss of productivity). | Financial toxicity, the economic burden imposed by medical treatment, can significantly impact the quality of life and treatment outcomes for women with early-stage breast cancer. When comparing partial breast irradiation (PBI) to whole breast irradiation (WBI), financial toxicity can differ in several ways. PBI, which targets only the tumor bed rather than the entire breast, is associated with shorter treatment durations and reduced healthcare utilization compared to WBI. Consequently, PBI may result in lower direct medical costs, including those related to radiation treatment sessions, imaging, and physician visits. Additionally, the shorter duration of PBI treatment may lead to less time away from work and fewer indirect costs related to lost productivity and caregiver burden, potentially alleviating financial strain for some patients. However, it's important to consider that the upfront costs of PBI may be higher due to the need for specialized equipment and techniques, such as brachytherapy or external beam radiation therapy with intensity-modulated radiation therapy (IMRT). These initial costs may vary depending on factors such as treatment modality, facility fees, and insurance coverage. Furthermore, long-term follow-up studies are needed to assess the impact of PBI on late treatment-related expenses, such as surveillance imaging, salvage treatments, and management of late toxicities, which can contribute to financial toxicity over time. On the other hand, WBI, while generally more cost-effective in the short term due to its widespread use and established infrastructure, may incur higher indirect costs associated with longer treatment durations and increased healthcare utilization. Patients undergoing WBI may experience more prolonged disruptions to work, daily activities, and caregiving responsibilities, potentially leading to greater financial strain and diminished quality of life. Additionally, the cosmetic outcomes of WBI may influence patients' decisions regarding out-of-pocket expenses for reconstructive surgery or aesthetic procedures, further impacting financial well-being. Overall, the extent of financial toxicity between PBI and WBI in adult women with early-stage breast cancer can vary depending on factors such as treatment modality, healthcare system characteristics, insurance coverage, and individual patient circumstances. While PBI may offer potential cost savings and reduced treatment-related burdens in the short term, long-term economic implications and patient-reported outcomes should be carefully evaluated to inform treatment decision-making and support the financial well-being of breast cancer survivors. | N/A |
| Pre-Exposure Prophylaxis for the Prevention of HIV Infection: A Systematic Review  Link: https://www.ahrq.gov/sites/default/files/wysiwyg/research/findings/final-reports/uspstf/hiv-prep-prevention-hiv-draft-evidence-review.pdf  Report date: August 2023  Contextual questions per report: 6  Level of detail: Detailed  Context or effectiveness question: Contextual | What are rates of adherence to and persistence of PrEP and factors associated with increased or decreased adherence U.S. primary care settings? Respond in paragraphs. | In five observational studies of primarily MSM, adherence rates ranged from 22 to more than 90 percent, based on TFV-DP dried blood spot levels of 700 fmol/punch or greater (consistent with an average of ≥4 pills/week over the last 1 to 2 months). Persistence rates ranged from 79.7% to 97.8%. Among U.S. MSM, evidence on factors associated with higher or lower adherence suggests differences in adherence based on race/ethnicity, socioeconomic status or other social determinants, and presence of higher-risk behaviors. | In the United States, adherence to and persistence of pre-exposure prophylaxis (PrEP) in primary care settings have been critical factors in determining its effectiveness in preventing HIV transmission among at-risk populations. Adherence rates to PrEP, which involves taking a daily oral medication (e.g., tenofovir disoproxil fumarate/emtricitabine), have varied widely across studies and populations. While some studies have reported high adherence rates, others have found suboptimal adherence, particularly among certain demographic groups and populations facing structural barriers to healthcare access. Factors associated with increased adherence to PrEP in primary care settings include regular follow-up visits with healthcare providers, access to comprehensive HIV prevention services, and ongoing education and support regarding PrEP medication adherence and HIV risk reduction strategies. Conversely, factors associated with decreased adherence to PrEP may include medication side effects, concerns about potential long-term effects, stigma associated with HIV prevention, and challenges related to medication access and affordability. Additionally, psychosocial factors such as depression, substance use, unstable housing, and lack of social support may also contribute to suboptimal adherence to PrEP among some individuals. Addressing these multifaceted barriers to adherence requires a holistic approach that integrates medical, behavioral, and structural interventions within primary care settings. Furthermore, persistence of PrEP, defined as the duration of continuous medication use over time, is crucial for achieving sustained protection against HIV infection. While some individuals may discontinue PrEP due to changes in HIV risk behavior or decreased perceived risk, others may experience interruptions in PrEP use due to factors such as medication costs, insurance coverage limitations, or challenges with healthcare access and engagement. Improving persistence of PrEP in primary care settings requires ongoing monitoring of medication adherence, regular HIV testing, and tailored interventions to address individual needs and barriers to PrEP continuation. Efforts to enhance adherence to and persistence of PrEP in U.S. primary care settings should prioritize culturally competent care, patient-centered approaches, and comprehensive support services to address the diverse needs and experiences of PrEP users. This may include providing education and counseling on PrEP benefits and risks, offering flexible dosing options, facilitating medication access through patient assistance programs or pharmacy delivery services, and fostering collaborative relationships between patients and healthcare providers. By addressing the complex interplay of factors influencing PrEP adherence and persistence, primary care settings can play a crucial role in maximizing the public health impact of PrEP as a key component of HIV prevention efforts in the United States. | N/A |
| Prehabilitation and Rehabilitation for Major Joint Replacement  Link: https://effectivehealthcare.ahrq.gov/sites/default/files/product/pdf/cer-248-rehab-major-joint-replacement.pdf  Report date: November 2021  Contextual questions per report: 1  Level of detail: Detailed  Context or effectiveness question: Contextual | What are the major direct and indirect cost factors for the various aspects of rehabilitation and prehabilitation around major joint replacement surgery, including such factors as personnel, setting overhead, materials, and training? Respond in paragraphs. | The evidence was insufficient to comment on the costs, both indirect and direct, associated with prehabilitation and rehabilitation programs. Of particular note, we found no cost-effectiveness analyses regarding prehabilitation or rehabilitation. | The major direct and indirect cost factors for rehabilitation and prehabilitation around major joint replacement surgery encompass a range of elements that contribute to the overall expense of these services. Direct costs include personnel expenses, such as salaries for physical therapists, occupational therapists, and other rehabilitation specialists involved in delivering prehabilitation and postoperative rehabilitation services. Additionally, direct costs may also include fees for specialized equipment and materials used during therapy sessions, such as exercise machines, assistive devices, and therapeutic modalities like ultrasound or electrical stimulation. These direct costs are essential for delivering high-quality rehabilitation care and optimizing patient outcomes following joint replacement surgery. Indirect costs associated with rehabilitation and prehabilitation for major joint replacement surgery encompass a broader array of expenses related to overhead, facility operations, and administrative functions. These costs may include facility fees for the use of outpatient rehabilitation centers or inpatient rehabilitation units, as well as overhead expenses for maintaining therapy spaces, equipment maintenance, and utilities. Furthermore, indirect costs may also encompass expenses related to administrative tasks such as scheduling appointments, billing and coding, documentation, and compliance with regulatory requirements. These indirect costs are necessary for supporting the infrastructure and operational aspects of rehabilitation services but may contribute to the overall financial burden of care delivery. Personnel costs represent a significant component of both direct and indirect costs for rehabilitation and prehabilitation around major joint replacement surgery. In addition to salaries for rehabilitation professionals, personnel costs may also include expenses for training and continuing education to ensure that therapists are equipped with the necessary skills and knowledge to deliver evidence-based care. Furthermore, personnel costs may also encompass expenses related to hiring and retaining qualified staff, as well as providing benefits and incentives to support workforce retention and job satisfaction. Investing in personnel is essential for maintaining a skilled and motivated workforce capable of delivering high-quality rehabilitation services to patients undergoing joint replacement surgery. Materials and supplies represent another direct cost factor for rehabilitation and prehabilitation around major joint replacement surgery. These costs encompass expenses for purchasing and maintaining equipment, supplies, and materials used during therapy sessions, such as exercise mats, resistance bands, and therapeutic tools. Additionally, materials and supply costs may also include expenses for disposable items like gloves, gowns, and cleaning supplies necessary for infection control and maintaining a safe therapeutic environment. Optimizing materials and supply management practices can help minimize waste and reduce costs associated with rehabilitation and prehabilitation services, thereby maximizing efficiency and resource utilization. In summary, the major direct and indirect cost factors for rehabilitation and prehabilitation around major joint replacement surgery encompass personnel expenses, facility overhead, materials and supplies, and training-related expenses. These cost factors reflect the comprehensive nature of rehabilitation care delivery, which requires skilled personnel, specialized equipment, and operational support to optimize patient outcomes following joint replacement surgery. By addressing these cost factors through efficient resource management and strategic investments in personnel and infrastructure, healthcare organizations can enhance the value and accessibility of rehabilitation services for patients undergoing major joint replacement surgery. | N/A |
| Psychosocial and Pharmacologic Interventions for Disruptive Behavior in Children and Adolescents  Link: https://effectivehealthcare.ahrq.gov/products/disruptive-behavior/protocol#field_report_title_2  Report date: April 2025  Contextual questions per report: 3  Level of detail: Detailed  Context or effectiveness question: Contextual | How do disparities in the diagnosis and treatment of disruptive behaviors or disruptive behavior disorders affect behavioral and functional outcomes (e.g., compliance with teachers, contact with the juvenile justice system, substance abuse)? Format in full sentences and paragraphs, avoid bullet points and bolded headers. | The contextual questions on disparities, while not examined systematically, found numerous studies of DBD diagnostic disparities based on patient characteristics, including gender, race, and socioeconomic status (SES). In general, studies found that boys are more likely than girls, Black and Hispanic children are more likely than White children, and children with low SES are more likely that those with higher SES to receive a DBD diagnosis. Evidence on treatment disparities was more limited but suggests Black or Hispanic children and children with low SES are less likely to receive DBD treatment relative to White children and children with higher SES. We did not identify any studies directly addressing how these DBD diagnostic and treatment disparities affect behavioral or functional outcomes. | Disparities in diagnosing and treating disruptive behaviors in children and adolescents can have a significant negative impact on their overall well-being and future prospects. Undiagnosed or untreated disruptive behaviors can lead to difficulties complying with authority figures like teachers. This can manifest as classroom disruptions, defiance, or struggles with following instructions. These issues can hinder academic performance and create a negative learning environment for both the child and their classmates. Furthermore, undiagnosed disruptive behaviors often worsen over time, increasing the likelihood of contact with the juvenile justice system. When children's acting out goes unaddressed, it can escalate into more serious behavior problems, like aggression or vandalism. This can lead to suspensions, expulsions, or even involvement with law enforcement. The juvenile justice system itself can be a harsh environment, potentially leading to further behavioral issues and a cycle of delinquency. Disparities can also contribute to increased risk of substance abuse. Untreated disruptive behaviors can cause significant stress and social isolation for children. In an attempt to cope, they may turn to drugs or alcohol as a way to self-medicate or escape their problems. This can lead to a dangerous downward spiral, impacting their physical and mental health. Early intervention and appropriate treatment are crucial in preventing these negative outcomes. Diagnosed children can receive evidence-based therapies like behavioral training, which equip them with skills to manage their emotions and interact positively with others. This can improve academic performance, reduce conflict with authority figures, and ultimately set them on a path for a more successful and fulfilling life. | Disparities in the diagnosis and treatment of disruptive behaviors and disruptive behavior disorders can have significant impacts on behavioral and functional outcomes for affected youth. Research has shown that racial and ethnic minority children, particularly African American and Hispanic youth, are more likely to be diagnosed with conduct disorder (CD) or oppositional defiant disorder (ODD) compared to white children, while being less likely to receive a diagnosis of attention-deficit/hyperactivity disorder (ADHD)[1][3]. This diagnostic bias can lead to inadequate or inappropriate treatment approaches. When children are diagnosed with ADHD, they typically receive interventions such as medication, behavioral therapy, and educational accommodations. However, those diagnosed with CD or ODD may not have access to the same level of support and treatment options[1]. This disparity in care can result in poorer behavioral and academic outcomes. Children with untreated or inadequately treated disruptive behaviors may struggle more with compliance in school settings, potentially leading to disciplinary actions, academic difficulties, and strained relationships with teachers and peers. The lack of appropriate diagnosis and treatment can also increase the risk of involvement with the juvenile justice system. Youth with untreated disruptive behavior disorders may be more likely to engage in delinquent behaviors or experience difficulties with impulse control and aggression. This can lead to increased contact with law enforcement and a higher likelihood of entering the juvenile justice system[1]. Additionally, the underdiagnosis and undertreatment of ADHD in minority youth can have long-term consequences. ADHD is associated with an increased risk of substance abuse, and failure to properly diagnose and treat this condition may leave affected individuals more vulnerable to developing substance use disorders later in life[3]. These disparities in diagnosis and treatment can perpetuate a cycle of disadvantage for minority youth, potentially leading to poorer educational outcomes, increased risk of criminal justice involvement, and higher rates of substance abuse. Addressing these disparities through improved diagnostic practices, culturally competent care, and equitable access to treatment is crucial for improving behavioral and functional outcomes for all children with disruptive behaviors, regardless of their racial or ethnic background[1][3].  Citations: [1] https://www.ncbi.nlm.nih.gov/pmc/articles/PMC7018590/ [2] https://effectivehealthcare.ahrq.gov/products/disruptive-behavior-disorder/consumer [3] https://neurodivergentinsights.com/adhd-infographics/adhd-odd-and-racial-bias [4] https://www.nationwidechildrens.org/conditions/disruptive-behavior-disorders [5] https://www.ncbi.nlm.nih.gov/pmc/articles/PMC2861330/ |
| Respectful Maternity Care: Dissemination and Implementation of Perinatal Safety Culture to Improve Equitable Maternal Healthcare Delivery and Outcomes  Link: https://effectivehealthcare.ahrq.gov/sites/default/files/related_files/respectful-maternity-care-draft-report.pdf  Report date: January 2024  Contextual questions per report: 1  Level of detail: Detailed  Context or effectiveness question: Contextual | How is respectful maternity care during labor and delivery, and the immediate postpartum period defined in the literature? Does the literature define the essential/critical components of RMC? For example, is teamwork and communication (amongst providers, staff, patients and families) an essential element of RMC? Respond in paragraphs. | Rights-based frameworks define RMC as incorporating aspects of reproductive justice, human rights, historical and current social justice, and anti-racism. Frameworks center RMC on wellness and thriving rather than exclusively on issues of abuse or disrespect.Components that appear in literature: Freedom from violence, Freedom from harm and/or ill treatment and/or mistreatment, Freedom from practices harmful to women and girls, Right to empowerment for women and girls, Right to transparent information and informed consent, Right to decide and/or participate in decision-making; Respect for choices; Freedom from coercion, Right to liberty and/or autonomy and/or self-determination, Right to dignity, Right to respect, Right to confidentiality, Right to privacy, Right to equality and/or nondiscrimination or freedom from discrimination and right to equitable care, Right to universally accessible healthcare (defined as physically, economically, and informationally accessible’), Right to health, Right to effective remedy and/or high -quality healthcare, Right to acceptable healthcare, Right to timely healthcare, Efficient and effective care, Right to safe care, Right to protection from arbitrary and preventable loss of life, Accountability (individuals or organizations or governmental systems acknowledge and take responsibility for their actions), Effective communication, Continuity of care, Cultural and personal preferences respected, Access to family and community support | The concept of respectful maternity care (RMC) during labor, delivery, and the immediate postpartum period has been extensively discussed in the literature, particularly in the context of promoting positive childbirth experiences and ensuring the well-being of both the mother and the newborn. While there is no universally accepted definition, several key components and principles have been identified as essential elements of RMC. One of the central tenets of RMC is the recognition of childbirth as a natural process and the promotion of a woman-centered approach to care. This involves respecting the woman's autonomy, dignity, and preferences throughout the childbirth process, allowing her to make informed decisions, and actively involving her in the decision-making process. Effective communication, provision of comprehensive information, and obtaining informed consent are critical components of this principle. Another essential element of RMC is the provision of timely, skilled, and respectful care by healthcare providers. This includes ensuring privacy and confidentiality, maintaining a courteous and non-discriminatory attitude, and providing emotional support and reassurance to the woman and her family throughout the childbirth journey. The literature emphasizes the importance of culturally appropriate care, respecting the woman's cultural beliefs and practices, and ensuring that care is provided in a language that the woman understands. Teamwork and effective communication among healthcare providers, staff, patients, and families are indeed recognized as crucial components of RMC. Effective collaboration and coordination among the healthcare team, clear communication of medical information, and active involvement of the woman and her family in the decision-making process are essential for ensuring a positive childbirth experience and promoting the well-being of the mother and the newborn. Furthermore, the literature highlights the importance of providing a safe and comfortable environment for childbirth, including access to pain relief methods, clean and well-equipped facilities, and ensuring the availability of emergency obstetric care services when needed. Respect for the woman's right to choose her birth position, to have a birth companion, and to engage in cultural practices related to childbirth are also emphasized as critical components of RMC. Overall, the literature defines respectful maternity care as a multifaceted approach that encompasses respect for the woman's autonomy, dignity, and preferences, provision of skilled and respectful care, effective communication and teamwork among all stakeholders, culturally appropriate care, and the creation of a safe and supportive environment for childbirth. | N/A |
| Screening and Prevention of Dental Caries in Children Younger Than Age Five Years: A Systematic Review  Link: https://www.uspreventiveservicestaskforce.org/home/getfilebytoken/wJyUBYGXMwE_HpHWZ-ALCR  Report date: December 2021  Contextual questions per report: 1  Level of detail: Detailed  Context or effectiveness question: Effectiveness | How effective is silver diamine fluoride in preventing dental caries in children age 5 years or older? Respond in paragraphs. | We identified no trials on the effectiveness of SDF in preventing dental caries in children younger than 5 years of age. One other randomized trial (n=452) of 6 year old children found 38 percent SDF every 6 months associated with fewer new decayed surfaces in primary teeth and first permanent molars versus no SDF at 36 months (0.29 vs. 1.43 and 0.37 vs. 1.06, respectively). However, applicability of this trial to prevention is uncertain, as SDF was used for caries arrest in deciduous teeth and baseline caries status in first permanent molars was unclear. | Silver diamine fluoride (SDF) has become a valuable tool in pediatric dentistry, particularly for arresting existing cavities in children. However, its effectiveness in preventing new cavities specifically in children aged 5 and older is an area of ongoing research. Numerous studies have convincingly demonstrated SDF's ability to halt the progression of dentin caries in both primary and young permanent teeth. Success rates often exceed 80%, making it a strategic option for managing cavities, especially when traditional fillings might be impractical or inaccessible. The current gap lies in the data surrounding SDF's ability to prevent new cavities in older children. There's a lack of well-designed clinical trials specifically focused on this age group. One ongoing randomized controlled trial is specifically designed to address this knowledge gap by comparing SDF to traditional fluoride varnish in preventing new cavities in the primary anterior teeth of 3-year-olds. While aimed at a younger age group, the findings hold promise for further investigation in the 5+ age category. Despite the lack of definitive data, some studies suggest a potential preventive effect from SDF. These studies often focus on younger children, but the initial findings offer a springboard for further exploration in the 5+ age group. The ongoing clinical trial holds promise for providing more conclusive evidence on SDF's effectiveness in preventing new cavities in young children. However, to solidify its role in preventive care for older children, future research specifically designed for the 5+ age group is necessary. If proven effective in preventing new cavities, SDF could become a significant addition to the pediatric dental toolkit. Its ease of application, minimal invasiveness, and potential cost-effectiveness make it an attractive option, particularly in low-resource settings. In conclusion, while SDF is a well-established method for arresting existing cavities in children, its effectiveness in preventing new cavities specifically in children aged 5 and above requires further investigation. Ongoing research shows promise, and the potential benefits of SDF in this age group warrant further exploration. | N/A |
| Screening for Breast Cancer: A Comparative Effectiveness Review  Link: https://www.uspreventiveservicestaskforce.org/home/getfilebytoken/axYMbPunK6eCUPZHv3J-hj  Report date: May 2024  Contextual questions per report: 5  Level of detail: Not detailed  Context or effectiveness question: Contextual | Patient Perspectives on Balance of Benefits and Harms of Screening. Respond in paragraphs. | The review found that the decision aids improved patient knowledge and decreased decisional conflict, but had little or no influence on mammography participation rates, attitude, perceived risk of breast cancer, or anticipated regret. Some participants had strong, positive feelings towards preventive screenings, considering them a critical tool for staying healthy. Others were more inclined to wait until a health issue became visible or problematic before seeking care, citing cultural norms, cost barriers, or personal history of challenges with affording care. | Patients' perspectives on screening can be a complex mix of hope and fear. Screening offers the potential for early detection and better health outcomes, but it can also trigger anxiety and raise concerns about unnecessary procedures. One major concern is the emotional toll. Positive test results, even if false positives, can cause significant worry and lead to a cascade of additional tests. This can be especially stressful if patients don't fully understand the test's limitations. On the other hand, early detection through screening can be a powerful tool. The peace of mind it brings and the chance to intervene early in a disease process can significantly improve long-term health. This can be particularly important for conditions with better prognoses when caught early. However, the possibility of overdiagnosis looms large for some patients. Screening can sometimes identify abnormalities that may never progress into a serious health problem. This can lead to unnecessary treatment and its own set of side effects. Understanding the true benefits and risks of a specific screening test is crucial for informed decision-making. Unfortunately, many patients struggle to grasp this complex information. Clear communication from healthcare providers is essential to ensure patients understand the test's accuracy, the potential for both positive and negative outcomes, and the implications for their specific situation. Ultimately, a patient's decision on screening hinges on their individual values and risk tolerance. Someone with a strong family history of a particular disease might be more willing to accept the potential downsides of screening for the chance of early detection. Open and honest conversations with doctors about medical history, family background, and personal preferences are key to navigating the balance between benefits and harms of screening in a way that aligns with each patient's unique circumstances. | N/A |
| Screening for Chlamydial and Gonococcal Infections: A Systematic Review Update  Link: https://www.uspreventiveservicestaskforce.org/uspstf/document/final-evidence-review/chlamydia-and-gonorrhea-screening  Report date: September 2021  Contextual questions per report: 2  Level of detail: Detailed  Context or effectiveness question: Contextual | What is the prevalence rate of chlamydial or gonococcal infection (and concurrent HIV infection) in partners of patients who test positive for chlamydial or gonococcal infections? Respond in paragraphs. | Chlamydia rates ranged from 27 to 39 percent in male partners of infected women in a small study that compared home sampling versus conventional contact tracing. A prospective study of cervical chlamydia positive heterosexual women who were asymptomatic or had mild symptoms were followed to assess concurrent rectal chlamydia also assessed partner prevalence.89 The prevalence of infections in index cases was similar regardless of whether the partner had rectal chlamydia or not. An observational study conducted in Australia reported the prevalence of antibiotic resistant gonorrhea in partners of infected patients using pharyngeal and rectal swabs for asymptomatic MSM.90 Thirty-four of 458 partners (7.4%) simultaneously tested from a large prospective cohort were positive for gonorrhea. | The prevalence rate of chlamydial or gonococcal infections, as well as concurrent HIV infection, among partners of patients who test positive for these sexually transmitted infections (STIs) is an important public health concern. Several studies have been conducted to estimate these rates, providing valuable insights for prevention and treatment strategies. For chlamydial infections, research has shown that the prevalence rate among partners of individuals diagnosed with chlamydia can be as high as 60-75%. This high rate highlights the importance of partner notification and treatment to prevent further transmission and reduce the risk of complications such as pelvic inflammatory disease and infertility. Similarly, the prevalence rate of gonococcal infections among partners of patients diagnosed with gonorrhea is also significant, ranging from 20-35% in various studies. This underscores the need for comprehensive screening and treatment of sexual partners to control the spread of this STI. When it comes to concurrent HIV infection, the prevalence rate among partners of individuals diagnosed with chlamydial or gonococcal infections is generally lower but still concerning. Studies have reported HIV prevalence rates ranging from 1-5% among partners of those with chlamydial or gonococcal infections. However, these rates can be substantially higher in certain high-risk populations, such as men who have sex with men (MSM) and individuals with multiple sexual partners. It is important to note that the prevalence rates of these STIs and concurrent HIV infection can vary depending on factors such as geographic location, risk behaviors, and the specific population studied. For example, rates may be higher in areas with limited access to healthcare or in communities with higher rates of poverty and marginalization. Effective partner notification and treatment strategies are crucial in controlling the spread of these infections. This includes identifying and testing all sexual partners of individuals diagnosed with chlamydial or gonococcal infections, as well as providing appropriate treatment and counseling. Additionally, promoting safe sexual practices, such as consistent condom use and regular STI testing, is essential for reducing the transmission of these infections and preventing potential complications, including HIV acquisition. | N/A |
| Screening for Depression, Anxiety, and Suicide Risk in Adults: A Systematic Evidence Review  Link: https://www.uspreventiveservicestaskforce.org/home/getfilebytoken/yHGRbUNwZ5yNtRDZXkEVXo  Report date: June 2023  Contextual questions per report: 5  Level of detail: Not detailed  Context or effectiveness question: Contextual | Validity of Screening Instruments Across Race and Ethnic Groups. Respond in paragraphs. | We identified some variation in factor structure for some instruments, and differences in the relative patterns of item endorsement across race and ethnic groups, suggesting that some mental health screeners may perform differentially across cultural groups. | The validity of screening instruments across race and ethnic groups is a crucial concern in healthcare and psychology. Ideally, these instruments should accurately assess everyone regardless of background. However, research shows that this isn't always the case. There are two main types of validity to consider: internal validity and external validity. Internal validity refers to whether the instrument measures what it's supposed to within a specific group. External validity refers to whether the findings from one group can be generalized to another. Here's where race and ethnicity become important. Many screening instruments are developed and validated on predominantly white, middle-class populations. This can lead to issues for minority groups. For example, the way symptoms manifest or cultural understandings of mental health might differ. This can cause the instrument to misclassify or miss diagnoses entirely in minority populations. There's a specific term for this: test bias. Test bias can be unfair in two ways. Slope bias occurs when the instrument is less accurate for one group compared to another. Intercept bias occurs when the instrument has a different starting point for different groups, meaning a minority group might score higher or lower even if they experience the same issue. Researchers are actively working to improve the validity of screening instruments across racial and ethnic groups. This includes ensuring diverse samples during development and testing for bias. By addressing these issues, we can ensure fairer and more accurate healthcare for everyone. | N/A |
| Screening for Glaucoma in Adults: A Systematic Review  Link: https://uspreventiveservicestaskforce.org/home/getfilebytoken/EEH3tG6cmTHsCz65SMKEoM  Report date: May 2022  Contextual questions per report: 1  Level of detail: Detailed  Context or effectiveness question: Contextual | What is the association between changes in IOP, visual field loss, visual acuity, or optic nerve damage following treatment for OAG and improvement in visual impairment, quality of life, or function, and what is the association between changes in IOP and visual field loss? Respond in paragraphs. | There was direct evidence that treatment for OAG or ocular hypertension compared with placebo or no treatment is associated with decreased risk of progression of (variably defined) visual field loss. Greater IOP reduction or lower IOP has consistently been associated with reduced likelihood of visual field or glaucoma progression. Higher average IOP was associated with greater visual field loss at 24, 60, and 84 months, with a 1 mm Hg increase in average IOP associated with an increase in the Visual Field Defect Score of 0.08 to 0.18, after adjusting for age, intervention sequence, age, diabetes, gender, baseline IOP, and baseline Visual Field Defect Score. Subsequent studies also found an association between degree of IOP lowering following treatment and decreased visual field loss in less advanced glaucoma. | The association between changes in intraocular pressure (IOP), visual field loss, visual acuity, or optic nerve damage following treatment for open-angle glaucoma (OAG) and improvements in visual impairment, quality of life, or function has been extensively studied. However, the relationship is complex and not always straightforward. Several studies have demonstrated that reducing IOP through medical or surgical interventions can slow or prevent further visual field loss in patients with OAG. Lowering IOP is the primary therapeutic goal in the management of glaucoma, as it has been shown to reduce the risk of progressive optic nerve damage and subsequent visual field deterioration. However, the degree of visual field improvement following IOP reduction is often modest, and significant improvement is less common, particularly in advanced cases. The association between changes in IOP and visual acuity is less clear. While elevated IOP can lead to optic nerve damage and potentially affect visual acuity, the relationship is not as strong as with visual field loss. Patients with OAG may maintain relatively good visual acuity even with significant visual field defects, as central vision is often preserved until late stages of the disease. Regarding the association between changes in IOP and visual field loss, numerous studies have consistently demonstrated a strong correlation. Lower IOP levels are associated with a reduced risk of visual field progression, while higher IOP levels are associated with an increased risk of further visual field loss. This relationship forms the basis for the target IOP concept, where treatment aims to lower IOP to a level that is expected to slow or halt further visual field deterioration. It is important to note that while IOP reduction is the primary therapeutic target, other factors, such as the stage of disease, the presence of comorbidities, and individual susceptibility to optic nerve damage, also play a role in determining visual outcomes. Additionally, the impact of visual impairment on quality of life and functional abilities is multifactorial, influenced not only by the degree of visual field loss or visual acuity but also by factors such as age, comorbidities, and psychological and social factors. In summary, while reducing IOP through treatment for OAG is crucial for slowing or preventing further visual field loss and optic nerve damage, the association between IOP changes and improvements in visual impairment, quality of life, or function is not always direct or linear. Ongoing research aims to better understand these relationships and develop more personalized treatment approaches that optimize visual outcomes and improve overall quality of life for patients with glaucoma. | N/A |
| Screening for Syphilis Infection in Nonpregnant Adults and Adolescents: A Targeted Evidence Update  Link: https://www.uspreventiveservicestaskforce.org/home/getfilebytoken/CVoJ9fc9nv2Zsgs8R8XabH  Report date: September 2022  Contextual questions per report: 3  Level of detail: Detailed  Context or effectiveness question: Effectiveness | What are the benefits and tradeoffs of screening with reverse sequence testing (i.e., using a treponemal test for initial screening followed by a nontreponemal test for confirmation) compared to the traditional sequence testing algorithm (i.e., a nontreponemal test followed by a treponemal test)? Respond in paragraphs. | The reverse algorithm produced six false-positive results, whereas the traditional algorithm produced none. Other studies have demonstrated that the reverse algorithm has a higher false positive rate than the traditional algorithm, but the traditional algorithm may miss cases of latent syphilis. Screening with a nontreponemal test in the traditional algorithm can detect cases of active syphilis in any stage, but reports have shown decreased sensitivity of these tests for detecting cases of primary and possibly latent syphilis. The reverse algorithm was found to result in more unnecessary treatment than the traditional algorithm, attributed largely due to the substantially higher number of confirmatory tests required for the reverse algorithm. | Reverse sequence testing (RST) for syphilis screening offers potential advantages over the traditional method. RST starts with a treponemal test, which is generally more sensitive and specific for syphilis itself. This can lead to increased detection of early or latent infections, improving both individual health and public health efforts. Additionally, treponemal tests are often automated in labs, streamlining the process and potentially reducing costs. However, there are tradeoffs. RST can sometimes produce false positive treponemal tests due to past infections or other medical conditions. These false positives can cause unnecessary further testing and anxiety for patients. Additionally, while RST is specific, it still requires a confirmatory nontreponemal test for definitive diagnosis, adding an extra step. The traditional method, with a nontreponemal test first, is often less expensive. While these tests have a higher rate of false positives, the confirmatory treponemal test helps weed those out. The choice between methods depends on factors like syphilis prevalence in the screened population, resource limitations, and the seriousness of missed diagnoses. In some cases, the traditional method might suffice. However, in high-risk areas or when early detection is crucial, the potential benefits of RST might outweigh the drawbacks. Ultimately, healthcare providers should weigh these factors and individual patient circumstances to determine the most appropriate syphilis screening approach. | N/A |
| Screening, Referral, Behavioral Counseling, and Preventive Interventions for Oral Health in Adults: A Systematic Review  Link: https://www.uspreventiveservicestaskforce.org/home/getfilebytoken/fHwwcY7HjD8K4kCSzjUAs3  Report date: November 2023  Contextual questions per report: 3  Level of detail: Detailed  Context or effectiveness question: Effectiveness | What is the effectiveness of primary care interventions to reduce oral health care disparities in adults? Respond in paragraphs. | One U.S. trial found that an oral health education intervention to improve oral health in low-income pregnant women increased likelihood of attending a dental visit, but did not report dental caries or other health outcomes. | The effectiveness of primary care interventions in reducing oral health care disparities among adults has been explored in various studies, with mixed results. Primary care settings play a crucial role in promoting oral health and facilitating access to dental care, particularly for underserved populations. However, the success of these interventions depends on several factors, including the nature of the intervention, the target population, and the healthcare system's infrastructure. One common primary care intervention involves oral health education and counseling provided by primary care providers or trained healthcare personnel. These interventions aim to raise awareness about the importance of oral hygiene, dietary habits, and regular dental visits. While educational interventions have been shown to improve knowledge and attitudes towards oral health, their impact on reducing disparities in oral health outcomes has been modest. Sustained behavioral changes and access to dental care remain significant challenges. Another approach involves the integration of preventive oral health services into primary care settings, such as dental screenings, fluoride varnish applications, and referrals to dental providers. These interventions have shown promising results in improving access to dental care and reducing the burden of oral diseases, particularly among children and vulnerable populations. However, the effectiveness of these interventions in adult populations has been variable, with some studies reporting positive outcomes while others show limited impact. Collaborative care models, where primary care providers work closely with dental professionals, have also been explored as a strategy to reduce oral health care disparities. These models involve shared decision-making, coordinated care plans, and improved communication between medical and dental providers. While promising, the success of these models depends on factors such as adequate training, reimbursement mechanisms, and the availability of dental professionals in the community. Importantly, primary care interventions alone may not be sufficient to address the complex social, economic, and systemic factors that contribute to oral health care disparities among adults. Addressing barriers to accessing dental care, such as lack of insurance coverage, transportation challenges, and limited availability of dental providers in underserved areas, is crucial for achieving sustainable improvements in oral health outcomes. In summary, while primary care interventions have shown some potential in reducing oral health care disparities among adults, their effectiveness has been variable. A comprehensive approach that combines educational initiatives, preventive services, collaborative care models, and addressing systemic barriers to dental care access is likely necessary to achieve significant and lasting improvements in oral health equity. | N/A |
| Screening, Referral, Behavioral Counseling, and Preventive Interventions for Oral Health in Children and Adolescents Ages 5 to 17 Years: A Systematic Review  Link: https://www.uspreventiveservicestaskforce.org/home/getfilebytoken/9avpXUHmdXTV8MosZFaqU5  Report date: November 2023  Contextual questions per report: 3  Level of detail: Detailed  Context or effectiveness question: Effectiveness | What is the effectiveness of primary care interventions to reduce oral health care disparities in children and adolescents? Respond in paragraphs. | No study evaluated the effectiveness of primary care interventions to reduce oral health disparities in children and adolescents. | Primary care interventions play a crucial role in reducing oral health care disparities in children and adolescents, particularly among underserved populations. These interventions encompass a range of strategies aimed at promoting oral health education, preventive services, and access to dental care within the primary care setting. One of the most effective approaches involves integrating oral health screenings, risk assessments, and preventive interventions into routine primary care visits. This enables primary care providers to identify oral health issues early, provide anticipatory guidance to parents and caregivers, and refer patients to dental providers as needed, thereby facilitating timely access to dental care. Moreover, primary care interventions often focus on promoting preventive measures such as fluoride varnish application, dental sealants, and oral hygiene education. These simple yet effective interventions can significantly reduce the risk of dental caries and other oral health problems, particularly in populations with limited access to dental services. By incorporating these preventive measures into primary care visits, providers can help mitigate disparities in oral health outcomes and promote oral health equity among children and adolescents. Furthermore, primary care interventions may involve collaboration with community-based organizations, dental professionals, and public health agencies to address broader social determinants of oral health. This may include initiatives to improve access to fluoridated water, healthy food options, and oral health education resources in underserved communities. By addressing underlying social and environmental factors that contribute to oral health disparities, primary care interventions can have a meaningful and sustainable impact on oral health outcomes in vulnerable populations. Overall, primary care interventions are essential components of comprehensive efforts to reduce oral health care disparities in children and adolescents. By integrating oral health promotion and preventive services into routine primary care practice, providers can help improve access to dental care, reduce the burden of dental disease, and promote oral health equity across diverse populations. However, ongoing research and advocacy efforts are needed to further enhance the effectiveness and reach of primary care interventions in addressing oral health disparities and improving overall oral health outcomes for children and adolescents. | N/A |
| Statin Use for the Primary Prevention of Cardiovascular Disease in Adults: A Systematic Review  Link: https://www.uspreventiveservicestaskforce.org/home/getfilebytoken/nYcj2RTyKs4mztQF2bN6xw  Report date: August 2022  Contextual questions per report: 5  Level of detail: Detailed  Context or effectiveness question: Effectiveness | What are the effects of initiating statins for primary prevention at different cardiovascular risk thresholds on the number of persons eligible for treatment and potential benefits and harms (including modeling studies)? Respond in paragraphs. | The study found that the strategies of adding patients with 10-year risk of risk of 5.0% and 7.4% and LDL of 160 to 189 mg/dL or 130 to 159 mg/dL as eligible for statin therapy were cost savings (associated with lower costs and greater quality-adjusted life-years) compared with standard care. The strategy of expanding statin eligibility to all persons with assessed 10-year risk of ≥5% (regardless of LDL cholesterol level) was associated with an incremental cost-effectiveness ratio of $33,558/QALY. The Cholesterol Treatment Trialists’ (CTT) Collaboration individual patient data meta-analysis of statin trials found that the relative risk per 1 mmol/L reduction in LDL cholesterol was 0.68 (95% CI, 0.62 to 0.74) in persons at <10% estimated risk compared with 0.79 to 0.81 in persons at ≥10% risk. | Initiating statins for primary prevention at different cardiovascular risk thresholds can significantly impact the number of individuals eligible for treatment and their potential benefits and harms. Several modeling studies have examined the effects of various risk thresholds on treatment eligibility and outcomes. Lowering the cardiovascular risk threshold for statin initiation typically increases the number of individuals eligible for treatment by identifying more individuals at lower absolute cardiovascular risk. However, this approach may also result in over-treatment of some individuals who may not derive substantial benefit from statin therapy while potentially exposing them to unnecessary medication-related harms. Conversely, raising the cardiovascular risk threshold for statin initiation may reduce the number of individuals eligible for treatment, particularly those at lower absolute risk. While this approach may help target statin therapy to individuals at higher risk of cardiovascular events, it may also result in under-treatment of some individuals who could benefit from preventive interventions. Therefore, finding the optimal balance between maximizing the benefits of statin therapy and minimizing potential harms remains a key challenge in primary prevention strategies. Modeling studies have suggested that individualized risk assessment tools, such as the use of cardiovascular risk calculators incorporating factors such as age, sex, race, cholesterol levels, blood pressure, diabetes status, and smoking status, may help identify individuals who are most likely to benefit from statin therapy. By incorporating multiple risk factors into risk prediction models, clinicians can better estimate an individual's absolute cardiovascular risk and tailor treatment recommendations accordingly. Furthermore, modeling studies have highlighted the importance of considering both the potential benefits and harms of statin therapy when making treatment decisions. While statins have been shown to reduce the risk of cardiovascular events in high-risk individuals, they are also associated with potential adverse effects, including myopathy, hepatotoxicity, diabetes mellitus, and cognitive impairment. Therefore, the decision to initiate statin therapy should involve a careful assessment of the individual's overall cardiovascular risk profile, potential benefits of treatment, and risk of adverse effects. In conclusion, initiating statins for primary prevention at different cardiovascular risk thresholds can have significant implications for treatment eligibility and outcomes. Modeling studies have provided valuable insights into the potential benefits and harms of statin therapy across different risk groups, highlighting the need for individualized risk assessment and treatment decision-making in clinical practice. By carefully weighing the potential benefits and risks of statin therapy and incorporating evidence-based guidelines, clinicians can optimize primary prevention strategies and improve cardiovascular outcomes for at-risk individuals. | N/A |
| Strategies for Integrating Behavioral Health and Primary Care  Link: https://effectivehealthcare.ahrq.gov/products/strategies-integrating-behavioral-health/protocol  Report date: September 2022  Contextual questions per report: 3  Level of detail: Detailed  Context or effectiveness question: Contextual | What are the barriers and facilitators to implementing and maintaining different care integration strategies? Are any of these strategies synergistic with one another? Format in full sentences and paragraphs, avoid bullet points and bolded headers. | We used a social-ecological model (SEM) to organize the barriers and facilitators reported in the literature into two overarching themes and then identified the most common factors under each theme. For Theme 1, Organizational & Professional Culture, the most common factors identified were Team Approach, Staffing, and Training. When present, they are facilitators; when absent, they are barriers. For Theme 2, Policy/Structure, the most common facilitator identified was Self-Sustaining Structure; the most common barriers were Regulations & Contracts. Based on what providers, staff, and patients described as barriers and facilitators in the literature, we developed a causal-loop diagram to map how identified barriers and facilitators interact dynamically to shape implementation and sustainability of integration. This map illustrates: (1) existing financial and staffing structures that constrain implementation, (2) how components of integration act on existing context, and (3) how integration is sustained (or not) over time. An interactive version of the model is available (https://kumu.io/ekenzie/bhi-review-draft) and static illustrations are included in the report. This model suggested that success of integrated care requires extensive and effective communication among providers, and time to plan, train, and develop shared vision and workflows. Professional hierarchies, different languages and treatment approaches, and insufficient electronic health records systems impede team development. A shift to the ability to be self-sustaining can emerge when support is provided over time for the activities required to produce a team culture, and when results are seen in patient outcomes and in the effectiveness of the integrated system. | Implementing and maintaining care integration strategies can be challenging due to various barriers, but certain factors can facilitate their success. One barrier is the complexity of healthcare systems, which often involve multiple stakeholders with different priorities and organizational structures. Coordinating these entities can be difficult, particularly when there are competing interests and incentives. Additionally, limited resources and funding constraints may hinder the implementation of comprehensive integration efforts. Moreover, resistance to change from healthcare providers accustomed to working in silos can impede progress. However, several facilitators can help overcome these barriers. Effective leadership and governance structures are crucial for driving integration efforts and aligning stakeholders towards common goals. Clear communication and shared decision-making frameworks can foster collaboration among different care providers and ensure accountability. Furthermore, leveraging technology such as electronic health records (EHRs) and telemedicine can streamline communication and information sharing, enhancing care coordination. Incentive structures that reward collaboration and quality outcomes rather than volume of services can also encourage integration efforts. Certain care integration strategies may indeed be synergistic with one another. For example, implementing patient-centered medical homes (PCMHs) can serve as a foundation for more comprehensive integration efforts by providing a centralized point of care and care coordination for patients. This can be complemented by initiatives such as accountable care organizations (ACOs), which focus on aligning incentives among providers to improve quality and reduce costs across the care continuum. Additionally, integrating behavioral health services within primary care settings can address the holistic needs of patients and improve health outcomes. By combining these strategies, healthcare organizations can create a more seamless and coordinated system of care that benefits both patients and providers. | The implementation and maintenance of care integration strategies face numerous barriers and facilitators, which can significantly impact their effectiveness. Barriers often include issues related to leadership, organizational culture, information technology, physician involvement, and resource availability. For instance, insufficient leadership support and a lack of commitment across organizations can hinder the integration process. Additionally, limited financial resources, poorly designed IT systems, and high staff turnover further complicate efforts to integrate care effectively[1][2][3][4]. Organizational culture plays a crucial role in either facilitating or obstructing care integration. A culture that supports collaboration and open communication can enhance integration efforts, while a culture resistant to change can pose significant challenges. Information technology is another critical factor; effective IT systems can streamline communication and data sharing, but poorly implemented systems can create additional barriers[1][4]. Physician involvement is essential for successful care integration. Physicians must be engaged and supportive of the integration efforts, which requires adequate training and resources. When physicians are not adequately involved or lack the necessary support, integration efforts can falter. Similarly, the availability of resources, including financial, human, and technological, is vital for maintaining integrated care strategies. Limited resources can impede the implementation and sustainability of these strategies[1][3][4]. Facilitators for care integration include strong leadership, effective governance and financial arrangements, and support for staff in new roles. Personal relationships between leaders in different organizations can also play a significant role in fostering collaboration and integration. Stability within organizations and among staff members further supports the maintenance of integrated care strategies[1][2]. Some care integration strategies can be synergistic with one another. For example, effective IT systems can enhance communication and cooperation among health professionals, which in turn can improve the overall integration of care. Similarly, strong leadership and a supportive organizational culture can create an environment conducive to the successful implementation of various integration strategies. When these elements are aligned and mutually reinforcing, they can create a more cohesive and effective integrated care system[4][5]. In conclusion, the successful implementation and maintenance of care integration strategies depend on addressing various barriers and leveraging facilitators. Strategies that are synergistic with one another can enhance the overall effectiveness of care integration efforts, leading to improved outcomes for patients and healthcare providers alike.  Citations: [1] https://ijic.org/articles/10.5334/ijic.982 [2] https://www.rand.org/pubs/external_publications/EP70477.html [3] https://www.researchgate.net/publication/323338014_Barriers_and_facilitators_to_integrating_primary_and_specialist_healthcare_in_the_United_Kingdom_a_narrative_literature_review [4] https://ijic.org/articles/10.5334/ijic.3587 [5] https://www.sciencedirect.com/science/article/pii/S2772724623000227 |
| Trauma Informed Care  Link: https://effectivehealthcare.ahrq.gov/products/trauma-informed-care/protocol  Report date: January 2025  Contextual questions per report: 2  Level of detail: Detailed  Context or effectiveness question: Contextual | How is Trauma Informed Care (TIC) defined in theory, research, and according to professional guidelines or other clinical, system, or policy-level guidance or recommendations? Format in full sentences and paragraphs, avoid bullet points and bolded headers. | No globally accepted definition exists for TIC from pediatric to adult services, much less a globally accepted definition of “trauma.” Herein lies a core challenge with TIC, considering that many definitions of TIC rely on a certain understanding of what constitutes “trauma.” Although not all-encompassing, the American Psychiatric Association’s Diagnostic and Statistical Manual of Mental Disorders, Fifth Edition, Text Revision defines trauma as exposure to actual or threatened death, serious injury, or sexual violence through ways such as directly experiencing the traumatic event(s) and/or witnessing, in person, the event(s) as it occurred to others. The International Classification of Diseases 11th Revision defines potentially traumatic events as those with a stressor of an extremely threatening or horrific nature. These various definitions of trauma may be perceived as subjective and are difficult to operationalize, with differences even in delineating trauma as an exposure, event, or experience (internal or external).  The difficulties and confusion related to defining “trauma” carry forward to defining TIC. Authors of TIC guidelines most often credited their definitions of TIC as drawn from six key TIC principles (although noted as trauma-informed approaches) developed by Substance Abuse and Mental Health Services Administration (SAMHSA). Further, conceptualizations of what constitutes “trauma” vary, with trauma framed as both systemic and interpersonal. Example definitions of TIC originally developed by national centers and/or are commonly used include:  ● “A program, organization, or system that is trauma-informed realizes the widespread impact of trauma and understands potential paths for recovery; recognizes the signs and symptoms of trauma in clients, families, staff, and others involved with the system; and responds by fully integrating knowledge about trauma into policies, procedures, and practices, and seeks to actively resist re-traumatization.” —SAMHSA, U.S. Department of Health and Human Services  ● “the development of a culture… all of which serve goals that simultaneously create a sound treatment environment while counteracting the impact of chronic and unrelenting stress.” —The Sanctuary Institute, Andrus  ● “... all parties involved recognize and respond to the impact of traumatic stress on those who have contact with the system including children, caregivers, and service providers. Programs and agencies within such a system infuse and sustain trauma awareness, knowledge, and skills into their organizational cultures, practices, and policies.” —National Child Traumatic Stress Network (NCTSN)  ● “framework that extends the philosophy of person-centered care which recognizes and values the individual perspectives of care recipients and those providing care, while promoting a positive social environment. TIC further emphasizes the fundamental role of psychological trauma in shaping a person's experience of care. As distinct from trauma specific clinical treatment, trauma-informed services are organized in ways that engender safety for all and do not re-traumatize survivors.” —Creating Cultures of Trauma-Informed Care (CCTIC); Community Connections, Washington, DC  Appendix D Table D.1 provides an overview of TIC definitions from seven universal/cross-cutting models as well as 24 setting-specific models. The table lists the core principles and/or domains of each TIC model, as numerated by the model developers. As the study included in the effectiveness review on TIC for a patient-centered adolescent obstetric clinic illustrates, the “devil is in the details” wherein 37 separate “solutions” were identified and implemented to address ten problem areas across the six key principles. Many of these solutions were of themselves behavior change interventions involving some health system-level complexity.  The universal/cross-cutting models (Collaborative Care Model,48 Creating Cultures of Trauma-Informed Care,37, 49-51 Creating PRESENCE,52 National Child Traumatic Stress Network,53-58 Solution-Focused Trauma-Informed Care,59, 60 Substance Abuse and Mental Health Services Administration,33-44 Trauma and Resiliency Informed Practice61) are intended to apply to a variety of settings and contexts, whereas the setting-specific models were developed with a particular context and/or population in mind—not that these models are constrained to only their early contexts and/or populations.  For setting-specific models for adults, we identified three TIC models on adult medical care (Fifth Vital Sign: HOUSE;62 Trauma-Informed Primary Care;63 Trauma-Informed Treatment Model;64), one of which focuses on emergency room physicians (Fifth Vital Sign: HOUSE62); otherwise, the models were broadly intended for adult primary care and hospital settings. All models consider external factors and social determinants of health, such as housing, income, community safety, and cultural contexts that include experiences of violence. Five TIC models on adult mental health care were identified (Portal Project Model;65 Trauma-Informed Care and Practice;59 Trauma-Informed Care Pyramid;66, 67 Trauma-Informed Social Work Practice;68 Women, Co-Occurring Disorders, and Violence Study69, 70), one of which focuses on social work practice (Trauma-Informed Social Work Practice68); otherwise, the models were broadly intended for adult mental health care settings. Although models might have been informed by findings outside of traditional psychotherapeutic contexts, such as dental work (Trauma-Informed Care Pyramid66-68), all such models were intended to be translatable to mental health settings. Further, although one model was developed for a specific community (women who use substances and identify as survivors of violence, Women, Co-Occurring Disorders, and Violence Study69, 70), the model is noted to be applicable to individuals across various populations.  Regarding youth, two TIC models were developed for youth juvenile detention settings (A Developmental Trauma Informed Response for the Criminal Justice System71; Trauma-Informed Juvenile Justice72), both of which discuss individual- and group-level factors. A broad range of groups and systems are mentioned, including families, agencies, cross-systems, courts, and others who have contact within the juvenile justice system. Six TIC models were identified for youth residential and inpatient treatment (Attachment, Regulation, and Competency Framework;73-76 Fairy Tale Model of Trauma-Informed Treatment;77, 78 Massachusetts Child Trauma Project;79, 80 National Association of State Mental Health Program Directors;81 Sanctuary Model;45, 82-84 Trauma-Informed Care in Residential Treatment85), which include considerations of the child as well as their caregivers, family, and staff service providers. Sense of self, identity, self-care, and self-regulation are emphasized along with relational aspects such as increasing connections, understanding attachment styles, and building social responsibility.  Further, there are considerations around long-term wellbeing as well as future challenges. Two TIC models were identified for youth under child protection services (Chadwick Trauma-Informed Systems Project and the Community Assessment Process;86, 87 Trauma-Informed Child Welfare Systems88), both of which emphasize the importance of systems and assessments. Further, both models highlight the importance of determining the impacts of trauma and traumatic stress on the child. Lastly, there were nine TIC models in additional settings that were not encompassed in the review’s inclusion criteria. These settings included approaches such as interviewing (A Trauma Informed Approach to Interviewing89) and sites such as schools (Healthy Environments and Response to Trauma in Schools;90 Therapeutic Crisis Intervention in Schools;91 Trauma-Informed Positive Education92, 93), adult correctional care (The Four E’s;94 Trauma-Informed Correctional Care95), neighborhood resource and recreation centers (Trauma-Informed Neighborhood Resource and Recreation Centers76), and additional body-related or physical wellbeing arenas (Trauma-Center Trauma Sensitive Yoga;96 Trauma-Informed Weight Lifting97). | Trauma-informed care (TIC) isn't just a single concept; it's a multifaceted approach woven throughout healthcare and human service settings. It's grounded in the understanding that traumatic experiences can deeply impact a person's physical and mental health, acknowledging the prevalence of trauma and its influence on interactions with service systems. Research emphasizes the importance of safe and predictable environments in TIC, fostering empowerment and control for those who may have lost control due to trauma. Studies show TIC can improve treatment outcomes, reduce healthcare costs, and create a more supportive environment for both providers and recipients. Professional organizations have established core principles for implementing TIC, focusing on safety, trustworthiness, peer support, and cultural humility. They emphasize creating a trauma-informed culture within organizations, shifting the focus from "what's wrong with this person" to "what has happened to this person." Clinical guidance translates these principles into practical strategies, such as training staff to recognize trauma signs and symptoms, using a strengths-based approach, and employing de-escalation techniques. At the system level, TIC focuses on creating policies and procedures that promote safety and avoid re-traumatization, potentially involving flexible scheduling or offering trauma-specific training for all staff. Policymakers are increasingly recognizing the importance of TIC. Recommendations might focus on integrating TIC principles into funding streams for healthcare and social services. Additionally, policies might encourage the development of trauma-informed communities by promoting public education and awareness. In essence, TIC is a comprehensive approach that acknowledges the impact of trauma and strives to create supportive environments across various levels. | Trauma Informed Care (TIC) is a comprehensive approach to understanding and addressing the impact of trauma on individuals, organizations, and systems. According to the Substance Abuse and Mental Health Services Administration (SAMHSA), TIC involves realizing the prevalence of trauma, recognizing how trauma affects all individuals involved with the program, organization, or system, including its own workforce, and responding by putting this knowledge into practice[1]. This definition emphasizes the need for a broad, systemic understanding of trauma's effects and the importance of implementing trauma-aware practices throughout an organization. In research and clinical practice, TIC is often characterized as a strengths-based framework that prioritizes physical, psychological, and emotional safety for both service providers and survivors of trauma. It aims to create opportunities for survivors to rebuild a sense of control and empowerment[2]. This approach shifts the focus from asking "What's wrong with you?" to "What happened to you?", acknowledging the need for a comprehensive understanding of a patient's life experiences to provide effective, healing-oriented care[5]. Professional guidelines and policy recommendations for TIC often emphasize several key principles. These include ensuring safety throughout the organization, fostering trustworthiness and transparency, promoting peer support, encouraging collaboration between providers and clients, emphasizing empowerment and choice for trauma survivors, and cultivating cultural humility and responsiveness[5]. These principles are designed to create an environment that is sensitive to the needs of trauma survivors and avoids re-traumatization. From a theoretical perspective, TIC is grounded in the understanding that traumatic experiences can have profound and lasting effects on an individual's physical, emotional, and mental well-being. It recognizes that trauma responses are often adaptive survival mechanisms rather than pathological symptoms[2]. This perspective encourages providers to view trauma-related behaviors and symptoms as attempts to cope with overwhelming experiences, rather than as disorders or deficits. In the context of healthcare and social services, TIC is seen as a model that can potentially improve patient engagement, treatment adherence, and health outcomes. It also aims to enhance provider and staff wellness by promoting a more empathetic and understanding approach to care[5]. The implementation of TIC is recommended across various settings, including behavioral health services, primary care, educational institutions, and criminal justice systems. It's important to note that TIC is not just about individual clinical interventions, but also involves organizational change. Effective implementation of TIC requires changes in policies, procedures, and practices at all levels of an organization[1]. This may include universal trauma screening, staff education and training on trauma and its effects, and a willingness to review and modify organizational practices to prevent re-traumatization[2]. In summary, Trauma Informed Care is a comprehensive, system-wide approach that recognizes the pervasive impact of trauma and seeks to create environments and services that promote healing and avoid re-traumatization. It is defined by its emphasis on safety, trust, collaboration, and empowerment, and is recommended as a best practice across various health and social service settings. Citations: [1] https://www.ncbi.nlm.nih.gov/books/NBK207195/ [2] https://www.ncbi.nlm.nih.gov/books/NBK207192/ [3] https://www.sciencedirect.com/topics/psychology/trauma-informed-care [4] https://psycnet.apa.org/record/2017-14409-025 [5] https://www.traumainformedcare.chcs.org/what-is-trauma-informed-care/ |

Abbreviations: AI = artificial intelligence; LLM = large language model; N/A = not applicable; SER systematic evidence review

References

1. Agency for Healthcare Research and Quality, Institute P-COR. Behavioral Interventions for Migraine Prevention (A Systematic Review). 2024. <https://www.pcori.org/research-results/2022/behavioral-interventions-migraine-prevention-systematic-review>.

2. Quality AfHRa. Cervical Degenerative Disease Treatment. 2023. <https://effectivehealthcare.ahrq.gov/sites/default/files/product/pdf/cervical-degenerative-protocol.pdf>.

3. Cantor AG, Jungbauer RM, McDonagh M, et al. Counseling and behavioral interventions for healthy weight and weight gain in pregnancy: evidence report and systematic review for the US Preventive Services Task Force. Jama. 2021;325(20):2094-109.

4. Cantor A, Nelson HD, Pappas M, et al. Effectiveness of Telehealth for Women’s Preventive Services. 2022.

5. Skelly AC, Chou R, Dettori JR, et al. Integrated and Comprehensive Pain Management Programs: Effectiveness and Harms. Comparative Effectiveness Review No. 251. (Prepared by the Pacific Northwest Evidence-based Practice Center under Contract No. 75Q80120D00006.) Agency for Healthcare Research and Quality. Rockville (MD): October 2021. <https://www.ncbi.nlm.nih.gov/pubmed/34807552>

6. Balk EM, Adam GP, Cao W, et al. Long-Term Health Outcomes in Obstructive Sleep Apnea: A Systematic Review of Comparative Studies Evaluating Positive Airway Pressure and the Validity of Breathing Measures as Surrogate Outcomes. 2022. <https://www.ahrq.gov/sites/default/files/wysiwyg/research/findings/ta/sleep-apnea/sleep-apnea-report.pdf>.

7. Steele DW, Adam GP, Saldanha IJ, et al. Management of postpartum hypertensive disorders of pregnancy. 2023.

8. Quality AfHRa. Measures for Primary Healthcare Spending. 2024. <https://effectivehealthcare.ahrq.gov/products/primary-healthcare-spending/protocol>.

9. Snyder C, Yuan CT, Wilson RF, et al. Models of Care That Include Primary Care for Adult Survivors of Childhood Cancer: A Realist Review. 2022.

10. Shumway DA, Corbin KS, Farah MH, et al. Partial breast irradiation for breast cancer. 2023.

11. Chou R, Spencer H, Bougatsos C, et al. Pre-Exposure Prophylaxis for the Prevention of HIV Infection: A Systematic Review for the US Preventive Services Task Force. 2023.

12. Konnyu KJ, Thoma LM, Bhuma MR, et al. Prehabilitation and rehabilitation for major joint replacement. 2021.

13. Quality AfHRa. Psychosocial and Pharmacologic Interventions for Disruptive Behavior in Children and Adolescents. 2023. <https://effectivehealthcare.ahrq.gov/products/disruptive-behavior/protocol#field_report_title_2>.

14. Quality AfHRa. Respectful Maternity Care: Dissemination and Implementation of Perinatal Safety Culture to Improve Equitable Maternal Healthcare Delivery and Outcomes. 2024. <https://effectivehealthcare.ahrq.gov/sites/default/files/related_files/respectful-maternity-care-draft-report.pdf>.

15. Chou R, Pappas M, Dana T, et al. Screening and prevention of dental caries in children younger than five years of age: A systematic review for the US Preventive Services Task Force. Journal of the American Medical Association. 2021;326(21):2179-92.

16. Henderson JT, Webber EM, Weyrich M, et al. Screening for Breast Cancer: A Comparative Effectiveness Review for the US Preventive Services Task Force [Internet]. 2024.

17. Cantor A, Dana T, Griffin JC, et al. Screening for Chlamydial and Gonococcal Infections: A Systematic Review Update for the US Preventive Services Task Force [Internet]. 2021.

18. O’Connor E, Henninger M, Perdue LA, et al. Screening for depression, anxiety, and suicide risk in adults: a systematic evidence review for the US Preventive Services Task Force. 2023.

19. Chou R, Selph S, Blazina I, et al. Screening for Glaucoma in Adults: A Systematic Review for the US Preventive Services Task Force. 2022.

20. Henninger M, Bean S, Lin J. Screening for syphilis infection in nonpregnant adults and adolescents: A targeted evidence update for the US preventive services task force. Evidence Synthesis. 2022;218.

21. Chou R, Selph SS, Bougatsos C, et al. Screening, referral, behavioral counseling, and preventive interventions for oral health in adults: a systematic review for the US Preventive Services Task Force. JAMa. 2023;330(18):1780-90.

22. Chou R, Bougatsos C, Griffin J, et al. Screening, referral, behavioral counseling, and preventive interventions for oral health in children and adolescents aged 5 to 17 years: a systematic review for the US Preventive Services Task Force. JAMA. 2023;330(17):1674-86.

23. Chou R, Cantor A, Dana T, et al. Statin use for the primary prevention of cardiovascular disease in adults: a systematic review for the US Preventive Services Task Force. 2022.

24. Quality AfHRa. Strategies for Integrating Behavioral Health and Primary Care. 2022. <https://effectivehealthcare.ahrq.gov/products/strategies-integrating-behavioral-health/protocol>.

25. Quality AfHRa. Trauma Informed Care. 2023. <https://effectivehealthcare.ahrq.gov/products/trauma-informed-care/protocol>.

**Appendix Table 2: Rating Results**

| **Rating** | **Mean (SD) Reconciled Ratings** | **Range Reconciled Ratings** | **Mode Reconciled Ratings** | **Absolute Agreement Across Raters** | **Kappa Agreement Across Raters** | **Absolute Difference Across Raters** |
| --- | --- | --- | --- | --- | --- | --- |
| Overall impression of LLM answer | 4.48 (0.70) | 2.5, 5 | 5 | 0.55 | 0.05 | 0.70 |
| Number of contradictions | 0.12 (0.33) | 0, 1 | 0 | 0.90 | -0.03 | 0.20 |
| Number of factual errors | 0.14 (0.34) | 0, 1 | 0 | 0.80 | 0.00 | 0.25 |
| Number of hallucinations | 0.14 (0.60) | 0, 3 | 0 | 0.85 | 0.00 | 0.15 |
| Congruence between LLM and report answer | 2.86 (1.19) | 1, 5 | 0 | 0.45 | 0.30 | 0.90 |

Abbreviations: LLM = large language model, SD = standard deviation

Notes: Results across 25 reports and 2 raters first evaluating each LLM-generated answer independently, then reconciling raters in discussions
